# A novel proposal of a simplified bacterial gene set and the neo-construction of a general minimized metabolic network

**DOI:** 10.1038/srep35082

**Published:** 2016-10-07

**Authors:** Yuan-Nong Ye, Bin-Guang Ma, Chuan Dong, Hong Zhang, Ling-Ling Chen, Feng-Biao Guo

**Affiliations:** 1 Center of Bioinformatics, Key Laboratory for NeuroInformation of the Ministry of Education, University of Electronic Science and Technology of China, Chengdu, 610054, China; 2 School of Biology and Engineering, Guizhou Medical University, Guiyang, 550025, China; 3 College of Informatics, Huazhong Agricultural University, Wuhan 430070, China; 4 Center for Information in BioMedicine, University of Electronic Science and Technology of China, Chengdu, 610054, China

## Abstract

A minimal gene set (MGS) is critical for the assembly of a minimal artificial cell. We have developed a proposal of simplifying bacterial gene set to approximate a bacterial MGS by the following procedure. First, we base our simplified bacterial gene set (SBGS) on experimentally determined essential genes to ensure that the genes included in the SBGS are critical. Second, we introduced a half-retaining strategy to extract persistent essential genes to ensure stability. Third, we constructed a viable metabolic network to supplement SBGS. The proposed SBGS includes 327 genes and required 431 reactions. This report describes an SBGS that preserves both self-replication and self-maintenance systems. In the minimized metabolic network, we identified five novel hub metabolites and confirmed 20 known hubs. Highly essential genes were found to distribute the connecting metabolites into more reactions. Based on our SBGS, we expanded the pool of targets for designing broad-spectrum antibacterial drugs to reduce pathogen resistance. We also suggested a rough semi-*de novo* strategy to synthesize an artificial cell, with potential applications in industry.

A minimal gene set (MGS) is defined as the smallest possible gene set necessary and sufficient to maintain a living organism^1^. The MGS represents the infrastructure of a minimal cell and could be taken as a foundation for synthetic biology to create strains with desirable traits^2^,^3^. MGS research is biologically significant because of the following reasons: (i) it can further our understanding of the origin and evolution of life - for example, it can aid in determining the last universal common ancestor (LUCA)^1^,^4^; (ii) a pathogen’s MGS can guide the development of antibiotics^3^,^5^; (iii) reducing metabolic pathways and downsizing the genome could result in useful industrial strains^6^; and, most importantly, (iv) it allows the construction of a minimal genome that can be used as a basis for synthetic biology^3^,^7^,^8^,^9^. Numerous researchers have contributed to the study of determining MGS^10^. *Mycoplasma genitalium*, a free-living bacterium with the smallest gene repertoire among the organisms sequenced to date, is an ideal species for MGS research and synthetic biology^11^ and has become the first genome to be subjected to genome-scale gene essentiality screening^12^. Mushegian and Koonin pioneered identification of an MGS by cross-species comparison, and they defined the first MGS, which contains ~256 conserved genes shared by the Gram-negative bacterium *Haemophilus influenzae* and the Gram-positive bacterium *M. genitalium*^13^. Later, Gil and colleagues reported a core MGS of 206 genes, after performing a comprehensive study based on the comparative genomics analysis of all available reduced genomes and experimental essentiality studies published at that time^14^. A minimal metabolism chart was also proposed. The minimal metabolic machinery is that which is indispensable to sustain life. Many studies devoted to determining the minimal (or core) metabolic network have been published. Burgard and colleagues approximated the core metabolic reactions of *Escherichia coli* under different culture conditions^15^, and Pollack and colleagues determined a list of minimum enzymatic reactions by reviewing the metabolic activities of various *Mycoplasma* species^16^. Castellanos *et al*. modeled purine and pyrimidine metabolic pathways in a minimal *E. coli* cell^17^. Recently, Barve *et al*. identified 133 “absolutely superessential” reactions in the *E. coli* metabolic network^18^, and Yang *et al*. defined a core metabolic proteome in an *E. coli* model^19^. Gil and colleagues explored the stoichiometric consistency and some architectural properties of the minimal metabolic network proposed by them in 2004 on rich environment^20^. Recently, Gil *et al*. updated their MGS by adding some genes to improve cell viability and new genes for RNA processing and metabolism^21^,^22^.

Gil *et al*. stated that the research of MGS should consider gene essentiality^22^. In recent years, the increasing availability of sequenced genomes and experimentally determined essential genes have prompted an update of the MGS. Thus, this project aimed to develop a novel proposal of simplifying bacterial gene set to approximate a bacterial MGS by combining metabolic network construction^23^ with comparative genomics based on known essential genes. In the proposal, a simplified bacterial gene set (SBGS) that preserves both the self-reproduction and self-maintenance systems is determined. We believe that this work provides valuable information for drug design and is a useful reference for building a novel bacterial chassis in synthetic biology.

## Results and Discussions

### Obtaining the initial SBGS by comparative genomics and neo-construction of the metabolic network

In this work, we obtained the initial SBGS by comparative genomics and supplemented it by neo-construction of a bacterial approximately minimal metabolic network. Persistent essential genes (PEGs) can be considered the backbone genes for all bacterial organisms. PEGs can be considered the backbone genes for all bacterial organisms. As described in the methods, we gathered 611 PEGs from the CEG database (Cluster of Essential Genes)^24^ (Supplementary File S1). Among them, 598 (97.88%) and 508 (83.14%) genes were determined to be functionally similar to the genes in *E. coli* MG 1655 and *Bacillus subtilis* 168, respectively. In PEGs, 11 genes were annotated as “hypothetical proteins”. All cluster sizes of these hypothetical proteins were three or four. Six genes with cluster size = 3 were annotated as “putative function”. Genes with larger cluster size were annotated as defined function. In total, 594 (97.22%, 611-11-6) genes with definite functions were re-annotated and distributed in 22 subsystems (Supplementary Table S1).

In contrast to previous studies wherein homologous genes are required to be conserved in all reference species, we developed a new strategy named half-retaining, which requires the homologous genes to be present in more than one half of the referenced species to compile a highly persistent essential gene set (HPEGS). The HPEGS) with 248 essential genes that persist in more than half (cluster size ≥ 15/2 ≈ 8) of the species in the CEG database was obtained (Supplementary Table S2).

We used the bootstrap method to demonstrate the stability of half-retaining strategy. The results are shown in Supplementary Tables S3 and S4. Regarding the gene identity, the overlap of the genes between HPEGS_14_ and HPEGS ranged from 87% to 95.9% (mean = 91.7% and variance = 0.0009%). The absence of high-level clades led to the lowest overlap, as seen in group 2 (i.e., deleting *B. subtilis* 168 led to no firmicutes phylum in the reference data). The gene numbers in each HPEGS were compared, and the overlapping rates were higher than the gene content, which ranged from 88.5% to 96.2% (mean = 92.4% and variance = 0.0006%). Thus, the SBGS is basically invariable, and may only exhibit slight destabilization when the reference set is changed. On the contrary, previous proposals included in the MGS only those genes conserved in all the reference species and it approaches a null set when the number of reference species increases gradually. As an example, an MGS of 256 genes was obtained in previous work^25^, but the number of genes was drastically reduced to 63 when 100 genomes were compared and was reduced to zero when using 1000 genomes^25^. Our loose definition has the advantage that if the reference species are randomly (with no bias and covering most major lineages) selected, the gene set remains stable regardless of the number of reference species used.

Metabolism is essential for organisms to sustain life. To artificially synthesize a cell, the minimal metabolism of a bacterial cell should be considered. Therefore, we constructed an approximate MMN to define the core genes involved in metabolism in the MGS. The top-down approach in synthetic biology is frequently used to downsize the function of the object^26^. Researchers can identify the essential components and functions of a cell based on its minimal function.

Therefore, on the next step to construct SBGS, we submitted the 594 genes included in the PEG set into Model_SEED to define a bacterial general metabolic network (GMN1). The GMN1 contains 555 reactions and 324 genes. By adding 84 essential genes (participating in 120 reactions), a viable bacterial metabolic model (GMN2) was obtained that encompassed 408 genes and 675 reactions.

Ultimately, 251 genes were removed from GMN2, resulting in a minimized bacterial metabolic network (MMN) with 157 genes that were involved in 431 reactions (Supplementary Fig. S1, Supplementary Tables S5 and 6 and Supplementary File S2). We only deleted genes with a cluster size <8, as well as their associated reactions, when we minimized the network. Hence, several redundant reactions associated with genes of cluster size >8 remain in our proposed bacterial MMN, and the bacterial MMN is approximately minimal. The redundancy of these reactions could increase the robustness of the network.

Consequently, by neo-constructing the approximate bacterial MMN, we obtained a gene set of 157 genes. Among these, 91 overlap with the collection of HPEGS. Hence, a SBGS including 314 (248 + 157 − 91) genes was obtained by the union of HPEGS and genes associated with the approximate bacterial MMN (Supplementary Table S2).

**Figure 1:**
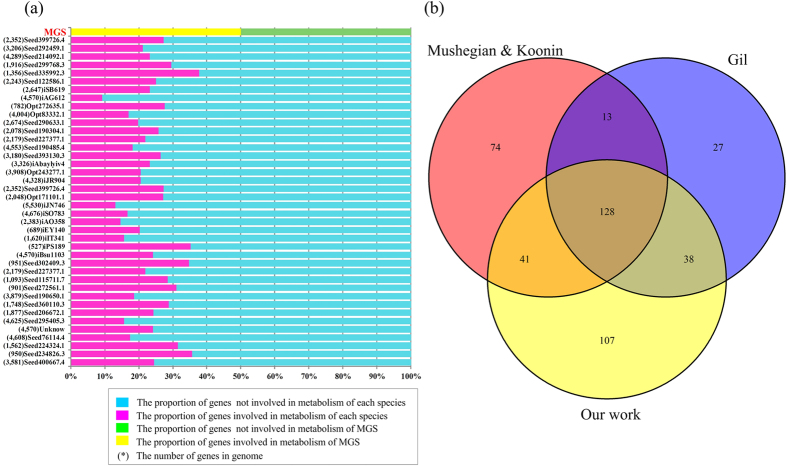
Comparison of the genes in our MGS and others. (**a**). The distribution of genes involved in the metabolism of each species and in the minimal gene set. (**b**) A Venn diagram for the three MGSs showing that our MGS contains 91% of the genes (128 of 141) existing in both Koonin *et al*.’s and Gil *et al*.’s MGSs, as well as our 107 newly identified genes (underlined in Supplementary Table S2).

We analyzed the gene proportions involved in the metabolism in 40 species; the resulting distribution is shown in Fig. 1a. We found that the value of the SBGS is close to the proportion of those species with small genomes in the reference genome list used.

In the SBGS, 66 genes with a cluster size <8 were added by constructing an approximate bacterial MMN, which corresponded to two extreme cases. The first is that the included genes have cluster sizes close to 8 (e.g., 7, 6 and 5; 38%). The second is that the included genes have cluster sizes close to 0 (e.g., cluster size = 0, 1, 2; 46%). The first case might be caused by the limitations of the techniques used to determine essential genes in difficult situations^27^ in which some factually essential genes might be overlooked in a few organisms. Genes functioning in a synergetic network may explain the second case. For example, three genes related to L-glutamine transport, *glnH*, *glnP* and *glnQ*, only have cluster sizes of 0 or 1. However, they all cooperate with the gene *glnS*, which has a cluster size of 8 and participates in the “GLNabcpp” reaction (Supplementary Table S5). The inactivation of *glnH*, *glnP* and *glnQ* may not interrupt the “GLNabcpp” reaction (thus resulting in a cluster size far less than 8); however, if the four genes *glnH*, *glnP*, *glnQ* and *glnS* are inactivated together, the “GLNabcpp” reaction is interrupted. Because the “GLNabcpp” reaction is of critical importance and has a super-essentiality = 1, the *glnS* gene is regarded as essential and the other three as nonessential. This reason leads to the existence of 18 nonessential genes in the bacterial MGS. However, they are involved in essential reactions of the approximate bacterial MMN. Kemmeren and Holstege confirmed that observing the effect of deleting a single gene might not precisely reveal all essential genes^28^ because the genes perform functions within a synergistic network (or function), and one defective gene may be substituted for by others performing similar functions.

After comparing our SBGS with previous MGS studies, we identified 169 and 166 genes that overlapped with sets reported by Mushegian and Koonin^13^. and Gil *et al*.^14^, respectively, as shown in Fig. 1b. Remarkably, our SBGS includes 91% (128) of the genes that exist in both of previous sets. The remaining 13 genes that exist in both Mushegian and Koonin’s and Gil *et al*.’s MGSs were lost in our SBGS (Supplementary Table S7). In order to ensure obtaining a SBGS as complete as possible, the genes existing in both Mushegian and Koonin’s and Gil *et al*.’s MGSs are considered as reliably essential in MGS. Hence, we added the 13 genes into our SBGS. As a result, our SBGS includes 314 + 13 = 327 genes. Moreover, we identified 107 new genes in our SBGS, including 62 genes identified by the half-retaining strategy and 45 genes identified using the approximate MMN construction method. Although our set might still be incomplete to perform all essential cell functions, the other two sets must be more affected by this issue because they were designed mainly based on ubiquitous genes. Henry and coworkers remarks that Gil *et al*.’s MGS omitted important genes involved in physiological processes, such as *dnaA* (replication initiation factor) and *dnaC* (a loading factor for helicase *dnaB*)^29^. The work of Henry *et al*. found that both Mushegian and Koonin’s and Gil *et al*.’s sets omitted 40 genes involved in RNA processing, metabolism and translation^29^. In contrast, our half-retaining strategy loosens the request of retaining in the final set and hence could contain more genes encoding essential functions. Supplementing it with those genes needed for a viable metabolic network makes our set more reliable although it may not be minimal.

We used COG (Clusters of Orthologous Groups)^30^ to categorize the functions of genes in different genes sets (Table 1). Table 1 demonstrates that the HPEGS covers 17 COG categories. Genes related to metabolism cover 17 COG categories, and two additional COG categories were added to the MGS (i.e., signal transduction mechanisms and defense mechanisms). After supplementing the genes by the neo-construction of an approximate bacterial MMN, and similarly to the Koonin’s proposal, our MGS includes 19 categories of COGs categories in total.

In summary, we have compiled a SBGS with 327 genes covering 19 COG categories sufficient to perform essential cellular functions by combining comparative genomics and metabolic network neo-construction. SBGS is a theoretical model and does not correspond to any existing species. The size of SBGS is consistent with the lower limit of theoretical gene numbers, as Koonin stated that the number of genes in the MGS would likely be in the range of 300–350 in nutrient-rich medium^4^.

Nevertheless, because MGS depends on the reference genomes used to extract it, there may not be a unique solution for a bacterial MGS. Our proposed SBGS provides an alternative reference for this issue.

### Comparisons with previous works and assigning essentialities to reactions in the approximate bacterial MMN

Supplementary Table S6 lists the number of reactions in each subsystem for our MMN. We compared them with the hypothetical minimal metabolism of Gil *et al*., and found that all reactions in Gil *et al*. exist in our MMN. Due to the half-retaining strategy, our approximate MMN has some redundant reactions that, as above stated, will increase the robustness of the network.

Barve *et al*. pioneered a method to rank reaction essentiality and proposed a concept of “super-essentiality” to estimate the importance of all reactions in the *E. coli* metabolic network^18^. The super-essentiality ranges from 0 to 1. One reaction with super-essentiality = 0 means that it is non-essential in the network. 133 absolutely super-essential reactions were identified in this manner. We selected the common reactions between their work and our approximate bacterial MMN model to analyze the essentiality of the reactions.

As a result (Supplementary Fig. S2, Supplementary Table S5), 261 reactions (60.56%) were categorized as super-essential. Among these, 213 reactions (81.61%) had super-essentiality values larger than zero, and 101 reactions had a value of one (absolutely super-essential reactions). The latter group included most of the absolutely super-essential reactions reported in Barve’s work, demonstrating the consistency of reaction essentiality between our approximate bacterial MMN model and Barve’s previous work. However, 32 absolutely super-essential reactions were absent from our approximate bacterial MMN. The difference between ours and Barve’s work might be caused by the different nutrient compositions of the medium. Barve e*t al*. identified super-essentialities based on different nutritional requirements, whereas we only used one type of medium (D-glucopyranose) when we performed Flux Balance Analysis (FBA).

### Determining key metabolites and genes by analyzing the topology of the bacterial approximately MMN and the application on drug targets development

We identified 25 key metabolites according to the node connectivity of metabolites in the approximate bacterial MMN (Table 2). Jeong *et al*. found that the hub metabolites were similar among all organisms^31^. In fact, most of the hub metabolites identified are “current metabolites”, as defined by Ma and colleagues^32^. Among the 25 key metabolites identified in this work, 14 are “current metabolites” suggested by both Ma *et al*.^32^ and Jeong *et al*.^31^, whereas six are consistent with Ma *et al*.^32^ only. The other five key metabolites are newly identified in this work.

Spearman correlation analysis showed that the node connectivity was significantly positively correlated with SBGS essentiality (*p* < 0.01, *rho* = 0.211; Table 3, see Methods). Both the average in-degree and out-degree of the metabolites were significantly negatively correlated with the bacterial MGS essentiality of a gene (*p* < 0.01, Table 3, see Methods). This result suggested that genes with a higher SBGS essentiality involve a lower average number of metabolites in a reaction.

Looking back in history of drug discovery, we found that the highly essential genes are often used as effective drug targets. For example, Haydon *et al*. synthesized an antibacterial drug based on *ftsZ*, a cell division protein that is present in SBGS with a cluster size = 14^33^. Recently, Ravishankar *et al*. identified a target for anti-tubercular, *topA* (cluster size = 10)^34^. Tharinjaroen *et al*. found a novel target, *lepB* gene (cluster size = 9), for *M. tuberculosis* and *M. bovis*^35^. Another example is the *fabG* gene (cluster size = 13), which is associated with 38 reactions and acts as a 3-ketoacyl-(acyl-carrier-protein) reductase. According to our results, the *fabG* gene may be a good candidate target for developing antibacterial drugs. To confirm this idea, we searched the DrugBank database and found that it had already been used as a drug target^36^. These successful examples indicate that highly essential genes in SBGS have been used in previous antibacterial drug discovery programs. Meanwhile, we find the highly essential genes often are hub nodes in approximate bacterial MMN.

Currently, the rapid emergence of multidrug-resistant pathogens has led to the ineffectiveness of conventional antibiotics for combating super bacteria^37^,^38^. Therefore, it is essential to develop new drugs to combat these pathogens. However, new drugs aimed at old targets may also meet with resistance. New drugs directed at new targets will be the most effective choice^37^. Considering that some targets are obsoleted prematurely, we must increase the pool of antibacterial drug targets^39^. We searched the DrugBank database for all genes in SBGS and found that 143 of 327 genes had been tagged as drug targets (Supplementary Table S8-1). This leaves 184 highly essential genes that have not yet been targeted by extant drugs (Supplementary Table S8-2). Among these, 102 genes do not have significant similarity with any human genes (BlastP E-value >10^−3^). These genes meet the most crucial criteria for broad spectrum antibacterial drug target selection: (1) a highly conserved function in a range of pathogens; (2) essentiality of the gene for the pathogens; and (3) no highly conserved function in humans^40^. Therefore, we suggest that these genes should be considered as targets in the development of new broad spectrum antibacterial drugs to expand the pool of targets for drug design.

### The application of SBGS on synthesis of an artificial cell

Foley and Shuler noted that engineers are interested in synthetic biology to develop a self-replicating biological system^41^. Pohorille *et al*.^42^ and Rasmussen *et al*.^43^ proposed that a human-made system could be considered “living” if three criteria are met: self-maintenance (metabolism), self-reproduction, and the capacity for Darwinian evolution. The approximate bacterial MMN informed the creation of our SBGS. Indeed, our SBGS includes abundant genes related to DNA replication, translation, transcription and posttranslational modification, protein turnover, and chaperones. Thus, it preserves both self-reproduction and self-maintenance systems.

**Figure 2:**
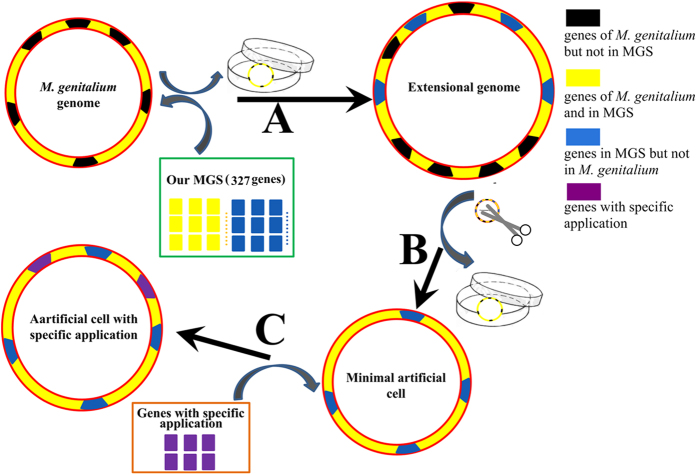
Our design for the semi-*de novo* synthesis of an artificial cell based on our MGS. (A) involves transferring the genes in the MGS but not in *M. genitalium* to the genome. (B) is knocking out the genes of *M. genitalium* that are absent from our MGS in the genome obtained in (A). (C) involves supplementing with genes required for specific applications.

Previously, scientists have synthesized artificial cells and chromosomes in the wet laboratory^44^,^45^,^46^ and have built computational simulation models^8^,^9^,^41^,^47^,^48^,^49^,^50^. Shuler’s group created the first mathematical model^50^ and recently developed a minimal cell model that can be tested by chemically simulating the behavior of a whole cell^9^. Scientists at the JCVI have tried to define a minimal *Mycoplasma* genome by gene deletion techniques^12^,^51^ and the chemically synthesized and assembled new a *Mycoplasma* genomes have been successfully introduced into a cell^45^,^46^,^51^. There are two strategies to synthesize artificial cells at present, top-down (genome downsizing) and bottom-up (*de novo* synthesis)^52^,^53^. Furthermore, Our SBGS could provide the third strategy for the synthesis of an applicable cell by the following procedure (Fig. 2). We suggest the semi-*de novo* synthesis of a cell, starting from *M. genitalium*. First, 101 genes of SBGS not present in *M. genitalium* would be integrated into its genome one by one (Supplementary Table S9). We would reference the gene order of the other species when transferring a new gene into the genome. After integrating one specific gene, we would verify the phenotype of the cell. If the cell does not thrive, we would abort the insertion of this gene into the genome. Thus, we would obtain an extensional genome with 475 + 101 = 576 genes. Second, we could knockout the 249 genes of *M. genitalium* that are absent in our MGS one by one from the extensional genome (Supplementary Table S10). Likewise, after knocking out a gene from the genome, we would determine its survival status. If the cell could not live normally, the gene would be retained. After completion of these steps, we would obtain an artificial cell with 327 genes that could live normally and could be regarded as a general bacterial chassis (Supplementary Table S11). Finally, we would supplement other genes into the chassis according to specific applications. Although it could provide a general reference of this issue, our proposal of semi-artificial bacterium might be vague in current form because the newly defined SBGS is just one additional theoretical proposal. A *Mycoplasma* has a quite special cell envelope and it may be not feasible to implement the proposal to simulate general metabolism that involves gram-positive and gram-negative bacteria. The minimal genome should not only include the MGS, but also contains the noncoding region such as noncoding RNA, UTR and gene control regions. For example, Serrano *et al*. have attempted to define the essential small ORFs and ncRNAs of a minimal cell^54^. Additionally, the gene order is highly variable among bacteria, and the chromosome architecture needs also to be taken into account. After all these points being addressed by us or the community in the near future, perhaps it will bring a novel bacterial species with great industrial applications.

## Methods

**Figure 3:**
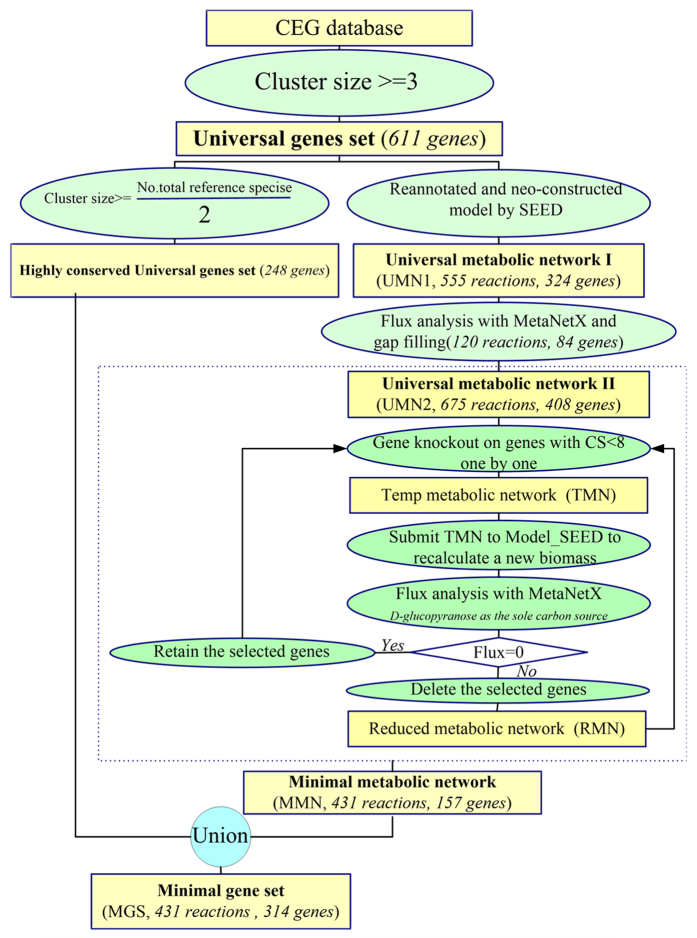
The procedure of this work.

Aiming at improving previous attempts to define a MGS, we proceeded through three steps (Fig. 3). First, we started from experimentally determined essential genes. Second, we developed a new strategy named half-retaining to identify essential genes conserved among over half of the reference species. Third, we supplemented our initial MGS of conserved essential genes by the neo-construction of a viable general metabolic network and subsequently downsizing it to an approximate minimal network.

### Data sources

The candidate essential genes were obtained from the CEG database^24^. The current version of CEG covers essential genes from 15 species of bacteria, which are listed in Supplementary Table S12. All clusters in the CEG correspond to essential genes for at least one reference species. Each cluster has a size value, indicating the number of reference species in which the corresponding gene is essential. Based on the cluster size, users can easily determine whether an essential gene is conserved in multiple species or is species-specific. Therefore, cluster size was used as a metric of conserved gene essentiality in the reference species and also of general gene essentiality in the SBGS. For example, the *pgsA* gene, with a cluster size of 15, is consistently essential in all 15 species. This result suggested that the *pgsA* gene is highly essential in the SBGS. The cluster size was used as a paramount index to determine the SBGS. To make the reconstructed model viable, we extracted a portion of the metabolic reactions from the iJR904 model^55^ and the iAF1260 model of *E. coli*^56^ (the two best annotated metabolic models) to fill the gaps in our reconstructed metabolic network.

### The half-retaining strategy

In contrast to previous studies wherein homologous genes are required to be conserved in all reference species, we developed a new strategy named half-retaining, which requires the homologous genes to be present in more than one half of the referenced species to compile a highly persistent essential gene set (HPEGS). In this work, a gene is considered a persistent essential gene (PEG) if it is essential and shared by more than three reference species. The PEG is evolutionarily conserved and serves as the basis for the SBGS for bacterial life. Thus, we determined an initial SBGS via comparative genomics, using a half-retaining strategy to compile a HPEGS.

We used the bootstrap method to demonstrate the advantage of this new approach. For 15 organisms, one was picked out each time, and the remaining 14 species were used as reference species. Thus, we generated 15 groups of reference species. For each group, we used the half-retaining strategy to obtain an HPEGS_14_ (a highly conserved universal gene set based on the 14 retained species). Subsequently, the new HPEGS_14_ was compared with the HPEGS obtained based on all 15 reference species.

### The construction of an approximate MMN

To construct a minimal metabolic network, the PEGs were re-annotated using the RAST tool^57^ of SEED^58^. The SEED annotator^57^ is based on the subsystems but not on sequence similarity. To estimate the reliability of the annotated genes, we compared them with *E. coli* MG1655 (Gram−) and *B. subtitlis* 168 (Gram+), which are two well-characterized model organisms, with the SEED tool. To downsize the genome and obtain an MGS with functional metabolic ability, we followed the procedure shown in Fig. 3.

First, GMN1 was neo-constructed based on the re-annotated PEG identified by the RAST tool of SEED. However, the GMN1 had gaps and was a dead network, lacking effective flux according to the FBA. The gaps were filled using the metabolic reactions extracted from the two metabolic models JR904 and iAF1260 of *E. coli*, which may be the most complete metabolic models thus far, until the viable (i.e., with effective flux in the biomass reaction) universal metabolic network GMN2 was obtained. To minimize the gene number, we deleted the disabled genes and disconnected reactions (i.e., those genes that could be knocked out and leave a viable network) from GMN2.

To refine the metabolic network according to the half-retaining strategy, genes with a cluster size ≥ 8 were reserved and regarded as the skeleton genes of the network, whereas those genes with a cluster size < 8 in GMN2 were deleted one by one in order of size. The temporary network was submitted to the MetaNetX website^59^ for flux analysis. We used growth medium with D-glucopyranose as the sole carbon source and rich in hydrogenphosphate, ammonium, water, proton, oxygen, and carbon dioxide for flux analysis. After single gene knockout from GMN2, if the flux is zero, the corresponding gene was retained in the network; otherwise it was dropped. New temporary networks were submitted to Model_Seed to regenerate a new biomass function to adapt to the new network.

### Calculation of topological properties of the defined metabolic network

To identify the key metabolites in the MMN, we used the total number of reactions affected by a metabolite as its connectivity. For example, 8 reactions used metabolite M as substrate or product; hence, the connectivity of M is 8. Attacking at the hub nodes could paralyze the network. Thus, the key metabolites play important roles in the network.

In addition to the analysis of key metabolites, key genes were also analyzed. We proposed a criterion that a gene is more essential in the MGS if it is essential in more individual genomes. Based on this criterion, we used “cluster size” in CEG to indicate the essentiality of a gene in the MGS. The number of reactions associated with a gene was defined as the node connectivity of the gene. Furthermore, to determine whether the average metabolite number of multiple reactions of a gene relates to its MGS essentiality, we analyzed the average in-degree and out-degree of the relevant reactions. For example, if a gene is associated with 5 reactions and its cluster size is 9. These 5 reactions have 25 substrates as well as 35 products totally. So its MGS essentiality is 9, its connectivity is 5, the average in-degree is 25/5 = 5 and average out-degree is 35/5 = 7.

## Additional Information

**How to cite this article**: Ye, Y.-N. *et al*. A novel proposal of a simplified bacterial gene set and the neo-construction of a general minimized metabolic network. *Sci. Rep*. **6**, 35082; doi: 10.1038/srep35082 (2016).

## Supplementary Material

Supplementary Information

## Figures and Tables

**Table 1:** The number of genes in each COG categories of different gene sets.

COG category	Number of gene in gene sets				
	HPEGS	MMN	SBGS	Koonin	Gil
Energy production and conversion (C)	12	13	16	17	9
Cell division and chromosome partitioning (D)	5	0	5	2	1
Amino acid transport and metabolism (E)	5	23	24	12	5
Nucleotide transport and metabolism (F)	13	15	14	17	15
Carbohydrate transport and metabolism (G)	11	17	18	15	17
Coenzyme metabolism (H)	14	19	19	8	12
Lipid metabolism (I)	25	28	29	4	7
Translation, ribosomal structure and biogenesis (J)	98	8	98	93	98
Transcription (K)	6	1	7	11	8
DNA replication, recombination and repair (L)	19	1	19	24	16
Cell envelope biogenesis, outer membrane (M)	20	18	25	8	2
Posttranslational modification, protein turnover, chaperones (O)	8	8	10	14	14
Inorganic ion transport and metabolism (P)	1	7	7	9	2
Secondary metabolites biosynthesis, transport and catabolism (Q)	3	3	3	1	0
General function prediction only (R)	13	1	13	11	0
Function unknown (S)	1	1	1	3	0
Signal transduction mechanisms (T)	0	2	2	1	2
Intracellular trafficking, secretion, and vesicular transport (U)	9	0	9	5	0
Defense mechanisms (V)	0	5	5	2	0

**Table 2:** Key metabolites in the network (out-degrees and in-degrees)^a^.

Metabolite name	Node connectivity	Metabolite description	Remarks
cpd00067	215	H	Current metabolite
cpd00001	122	H2O	Current metabolite
cpd11493	79	ACP	Current metabolite
cpd00002	73	ATP	Current metabolite
cpd00011	56	CO2	Current metabolite
cpd00008	54	ADP	Current metabolite
cpd00006	52	NADP	Current metabolite
cpd00005	49	NADPH	Current metabolite
cpd00012	45	PPi	Current metabolite
cpd00003	42	NAD	Current metabolite
cpd11492	40	Malonyl-acyl-carrierprotein	Ma
cpd00010	39	CoA	Ma
***cpd00009***	***38***	***Phosphate***	++
cpd00004	37	NADH	Current metabolite
cpd00080	33	Glycerol-3-phosphate	Ma
cpd00046	30	CMP	Current metabolite
cpd00052	19	CTP	Current metabolite
cpd00018	19	AMP	Current metabolite
***cpd00033***	***16***	***Glycine***	++
***cpd00054***	***15***	***L-Serine***	++
***cpd00035***	***11***	***L-Alanine***	++
***cpd10516***	***11***	***fe3***	++
cpd00023	10	L-Glutamate	Ma
cpd00020	9	Pyruvate	Ma
cpd00061	9	Phosphoenolpyruvate	Ma

^a^The key metabolites in bold and italics marked with “++” are newly found in this work. The metabolites marked as “Current metabolite” are consistent with the work of Ma *et al*. (Ma & Zeng, 2003) and of Jeong *et al*. (Jeong *et al*.^31^). The metabolites marked as “Ma” are consistent with the work of Ma (Ma & Zeng,^32^).

**Table 3:** Spearman correlation between MGS essentiality and node connectivity^a^.

Object	rho	p-value
Essentiality ~ node connectivity	0.211	0.008198
Essentiality ~ average in-degree	−0.274	0.000512
Essentiality ~ average out-degree	−0.281	0.000369
Essentiality ~ average degree	−0.278	0.000424

^a^MGS essentiality is represented by cluster size; node connectivity is represented by the number of reactions associated with a gene; average in-degree is represented by the number of reactants divided by that of reactions associated with a gene; average out-degree is represented by the number of products divided by that of reactions associated with a gene; average degree is represented by the number of metabolites divided by that of reactions associated with a gene.

## References

[b1] Koonin E. V. How many genes can make a cell: the minimal-gene-set concept. Annu Rev Genomics Hum Genet 1, 99–116 (2000). 11701626 10.1146/annurev.genom.1.1.99 PMC4780915

[b2] Davierwala A. P. . The synthetic genetic interaction spectrum of essential genes. Nature Genetics 37, 1147–1152 (2005). 16155567 10.1038/ng1640

[b3] Juhas M., Eberl L. & Church G. M. Essential genes as antimicrobial targets and cornerstones of synthetic biology. Trends in Biotechnology 30, 601–607 (2012). 22951051 10.1016/j.tibtech.2012.08.002

[b4] Koonin E. V. Comparative genomics, minimal gene-sets and the last universal common ancestor. Nature Reviews Microbiology 1, 127–136 (2003). 15035042 10.1038/nrmicro751

[b5] Roemer T. . Large-scale essential gene identification in Candida albicans and applications to antifungal drug discovery. Mol Microbiol 50, 167–181 (2003). 14507372 10.1046/j.1365-2958.2003.03697.x

[b6] Lee J. H. . Metabolic engineering of a reduced-genome strain of Escherichia coli for L-threonine production. Microb Cell Fact 8, 2 (2009). 19128451 10.1186/1475-2859-8-2 PMC2634754

[b7] Mushegian A. The minimal genome concept. Current Opinion in Genetics & Development 9, 709–714 (1999). 10607608 10.1016/s0959-437x(99)00023-4

[b8] Karr J. R. . A whole-cell computational model predicts phenotype from genotype. Cell 150, 389–401 (2012). 22817898 10.1016/j.cell.2012.05.044 PMC3413483

[b9] Shuler M. L., Foley P. & Atlas J. Modeling a minimal cell. Methods Mol Biol 881, 573–610 (2012). 22639227 10.1007/978-1-61779-827-6_20

[b10] Juhas M. On the road to synthetic life: the minimal cell and genome-scale engineering. Crit Rev Biotechnol, 1–8 (2015). 10.3109/07388551.2014.989423 25578717

[b11] Fraser C. M. . The minimal gene complement of Mycoplasma genitalium. Science 270, 397–403 (1995). 7569993 10.1126/science.270.5235.397

[b12] Glass J. I. Essential genes of a minimal bacterium. Proceedings of the National Academy of Sciences 103, 425–430 (2006). 10.1073/pnas.0510013103 PMC1324956 16407165

[b13] Mushegian A. R. & Koonin E. V. A minimal gene set for cellular life derived by comparison of complete bacterial genomes. Proc Natl Acad Sci USA 93, 10268–10273 (1996). 8816789 10.1073/pnas.93.19.10268 PMC38373

[b14] Gil R., Silva F. J., Pereto J. & Moya A. Determination of the Core of a Minimal Bacterial Gene Set. Microbiology and Molecular Biology Reviews 68, 518–537 (2004). 15353568 10.1128/MMBR.68.3.518-537.2004 PMC515251

[b15] Burgard A. P., Vaidyaraman S. & Maranas C. D. Minimal reaction sets for Escherichia coli metabolism under different growth requirements and uptake environments. Biotechnol Prog 17, 791–797 (2001). 11587566 10.1021/bp0100880

[b16] Pollack J. D., Williams M. V. & McElhaney R. N. The comparative metabolism of the mollicutes (Mycoplasmas): the utility for taxonomic classification and the relationship of putative gene annotation and phylogeny to enzymatic function in the smallest free-living cells. Crit Rev Microbiol 23, 269–354 (1997). 9439886 10.3109/10408419709115140

[b17] Castellanos M., Wilson D. B. & Shuler M. L. A modular minimal cell model: purine and pyrimidine transport and metabolism. Proc Natl Acad Sci USA 101, 6681–6686 (2004). 15090651 10.1073/pnas.0400962101 PMC404105

[b18] Barve A., Rodrigues J. F. & Wagner A. Superessential reactions in metabolic networks. Proc Natl Acad Sci USA 109, E1121–E1130 (2012). 22509034 10.1073/pnas.1113065109 PMC3345022

[b19] Yang L. . Systems biology definition of the core proteome of metabolism and expression is consistent with high-throughput data. Proc Natl Acad Sci USA 112, 10810–10815 (2015). 26261351 10.1073/pnas.1501384112 PMC4553782

[b20] Gabaldon T. . Structural analyses of a hypothetical minimal metabolism. Philosophical Transactions of the Royal Society B-Biological Sciences 362, 1751–1762 (2007). 10.1098/rstb.2007.2067 PMC2442391 17510022

[b21] Gil R. The Minimal Gene‐Set Machinery. Encyclopedia of Molecular Cell Biology and Molecular Medicine (2014).

[b22] Gil R. & Peretó J. Small genomes and the difficulty to define minimal translation and metabolic machineries. Frontiers in Ecology and Evolution 3, 123 (2015).

[b23] Burgard A. P., Nikolaev E. V., Schilling C. H. & Maranas C. D. Flux coupling analysis of genome-scale metabolic network reconstructions. Genome Res 14, 301–312 (2004). 14718379 10.1101/gr.1926504 PMC327106

[b24] Ye Y. N., Hua Z. G., Huang J., Rao N. & Guo F. B. CEG: a database of essential gene clusters. BMC Genomics 14, 769 (2013). 24209780 10.1186/1471-2164-14-769 PMC4046693

[b25] Acevedo-Rocha C. G., Fang G., Schmidt M., Ussery D. W. & Danchin A. From essential to persistent genes: a functional approach to constructing synthetic life. Trends Genet 29, 273–279 (2013). 23219343 10.1016/j.tig.2012.11.001 PMC3642372

[b26] Sabatier P. A. Top-down and bottom-up approaches to implementation research: a critical analysis and suggested synthesis. Journal of public policy 6, 21–48 (1986).

[b27] Wei W., Ning L. W., Ye Y. N. & Guo F. B. Geptop: a gene essentiality prediction tool for sequenced bacterial genomes based on orthology and phylogeny. PLoS ONE 8, e72343 (2013). 23977285 10.1371/journal.pone.0072343 PMC3744497

[b28] Kemmeren P. . Large-scale genetic perturbations reveal regulatory networks and an abundance of gene-specific repressors. Cell 157, 740–752 (2014). 24766815 10.1016/j.cell.2014.02.054

[b29] Henry C., Overbeek R. & Stevens R. L. Building the blueprint of life. Biotechnol J 5, 695–704 (2010). 20665643 10.1002/biot.201000076

[b30] Galperin M. Y., Makarova K. S., Wolf Y. I. & Koonin E. V. Expanded microbial genome coverage and improved protein family annotation in the COG database. Nucleic Acids Res 43, D261–D269 (2015). 25428365 10.1093/nar/gku1223 PMC4383993

[b31] Jeong H., Tombor B., Albert R., Oltvai Z. N. & Barabasi A. L. The large-scale organization of metabolic networks. Nature 407, 651–654 (2000). 11034217 10.1038/35036627

[b32] Ma H. & Zeng A.-P. Reconstruction of metabolic networks from genome data and analysis of their global structure for various organisms. Bioinformatics 19, 270–277 (2003). 12538249 10.1093/bioinformatics/19.2.270

[b33] Haydon D. J. . An inhibitor of FtsZ with potent and selective anti-staphylococcal activity. Science 321, 1673–1675 (2008). 18801997 10.1126/science.1159961

[b34] Ravishankar S. . Genetic and chemical validation identifies Mycobacterium tuberculosis topoisomerase I as an attractive anti-tubercular target. Tuberculosis (Edinb) 95, 589–598 (2015). 26073894 10.1016/j.tube.2015.05.004

[b35] Tharinjaroen C. S. . Novel Targeting, lepB Gene, Using Polymerase Chain Reaction with Confronting Two Pair Primers (PCR-CTPP) for Simultaneous Detection of Mycobacterium tuberculosis complex and Mycobacterium bovis. J Med Microbiol (2015). 10.1099/jmm.0.000188 26474823

[b36] Law V. . DrugBank 4.0: shedding new light on drug metabolism. Nucleic Acids Res 42, D1091–1097 (2014). 24203711 10.1093/nar/gkt1068 PMC3965102

[b37] Wilson D. N. Ribosome-targeting antibiotics and mechanisms of bacterial resistance. Nat Rev Microbiol 12, 35–48 (2014). 24336183 10.1038/nrmicro3155

[b38] Fischbach M. A. & Walsh C. T. Antibiotics for emerging pathogens. Science 325, 1089–1093 (2009). 19713519 10.1126/science.1176667 PMC2802854

[b39] Schmid M. B. Do targets limit antibiotic discovery? Nat Biotechnol 24, 419–420 (2006). 16601725 10.1038/nbt0406-419

[b40] Haselbeck R. . Comprehensive essential gene identification as a platform for novel anti-infective drug discovery. Curr Pharm Des 8, 1155–1172 (2002). 12052225 10.2174/1381612023394818

[b41] Foley P. L. & Shuler M. L. Considerations for the design and construction of a synthetic platform cell for biotechnological applications. Biotechnol Bioeng 105, 26–36 (2010). 19816966 10.1002/bit.22575

[b42] Pohorille A. & Deamer D. Artificial cells: prospects for biotechnology. Trends Biotechnol 20, 123–128 (2002). 11841864 10.1016/s0167-7799(02)01909-1

[b43] Rasmussen S. . Transitions from nonliving to living matter. Science 303, 963–965 (2004). 14963315 10.1126/science.1093669

[b44] Dymond J. S. . Synthetic chromosome arms function in yeast and generate phenotypic diversity by design. Nature 477, 471–476 (2011). 21918511 10.1038/nature10403 PMC3774833

[b45] Gibson D. G. . Creation of a bacterial cell controlled by a chemically synthesized genome. Science 329, 52–56 (2010). 20488990 10.1126/science.1190719

[b46] Gibson D. G. . Complete chemical synthesis, assembly, and cloning of a Mycoplasma genitalium genome. Science 319, 1215–1220 (2008). 18218864 10.1126/science.1151721

[b47] Takahashi K. . E-Cell 2: multi-platform E-Cell simulation system. Bioinformatics 19, 1727–1729 (2003). 15593410 10.1093/bioinformatics/btg221

[b48] Tomita M. . E-CELL: software environment for whole-cell simulation. Bioinformatics 15, 72–84 (1999). 10068694 10.1093/bioinformatics/15.1.72

[b49] Browning S. T. & Shuler M. L. Towards the development of a minimal cell model by generalization of a model of *Escherichia coli*: use of dimensionless rate parameters. Biotechnol Bioeng 76, 187–192 (2001). 11668452 10.1002/bit.10007

[b50] Shuler M., Leung S. & Dick C. A mathematical model for the growth of a single bacterial cell*. Annals of the New York Academy of Sciences 326, 35–52 (1979).

[b51] Hutchison C. A. . Design and synthesis of a minimal bacterial genome. Science 351, aad6253 (2016). 27013737 10.1126/science.aad6253

[b52] Esvelt K. M. & Wang H. H. Genome-scale engineering for systems and synthetic biology. Mol Syst Biol 9, 641 (2013). 23340847 10.1038/msb.2012.66 PMC3564264

[b53] Kosuri S. & Church G. M. Large-scale de novo DNA synthesis: technologies and applications. Nat Methods 11, 499–507 (2014). 24781323 10.1038/nmeth.2918 PMC7098426

[b54] Lluch-Senar M. . Defining a minimal cell: essentiality of small ORFs and ncRNAs in a genome-reduced bacterium. Mol Syst Biol 11, 780 (2015). 25609650 10.15252/msb.20145558 PMC4332154

[b55] Reed J. L., Vo T. D., Schilling C. H. & Palsson B. O. An expanded genome-scale model of Escherichia coli K-12 (iJR904 GSM/GPR). Genome Biol 4, R54 (2003). 12952533 10.1186/gb-2003-4-9-r54 PMC193654

[b56] Orth J. D. . A comprehensive genome-scale reconstruction of Escherichia coli metabolism–2011. Mol Syst Biol 7, 535 (2011). 21988831 10.1038/msb.2011.65 PMC3261703

[b57] Aziz R. K. . The RAST Server: rapid annotations using subsystems technology. BMC Genomics 9, 75 (2008). 18261238 10.1186/1471-2164-9-75 PMC2265698

[b58] DeJongh M. . Toward the automated generation of genome-scale metabolic networks in the SEED. BMC Bioinformatics 8, 139 (2007). 17462086 10.1186/1471-2105-8-139 PMC1868769

[b59] Ganter M., Bernard T., Moretti S., Stelling J. & Pagni M. MetaNetX.org: a website and repository for accessing, analysing and manipulating metabolic networks. Bioinformatics 29, 815–816 (2013). 23357920 10.1093/bioinformatics/btt036 PMC3597148

