# Supplementary material for: A novel proposal of a simplified bacterial gene set and the neo-construction of a general minimized metabolic network
Source: Sci Rep. 2016 Oct 7;6:35082. doi: 10.1038/srep35082 (PMC5054358; doi:10.1038/srep35082)
Supplement: Supplementary Information [file srep35082-s1.pdf]

Supplementary Information for

A novel proposal of a simplified bacterial gene set and the neo-construction of a general minimized metabolic network

Yuan-Nong Ye[1,2], Bin-Guang Ma[3], Chuan Dong[1,2], Hong Zhang[3], Ling-Ling Chen[3] and Feng-Biao Guo[1,2*]

1

Center of Bioinformatics, Key Laboratory for NeuroInformation of the Ministry of Education, University of Electronic Science and Technology of China, Chengdu, 610054, China

2

Center for Information in BioMedicine, University of Electronic Science and Technology of China, Chengdu, 610054, China

3

College of Informatics, Huazhong Agricultural University, Wuhan 430070, China.

*Corresponding author. E-mail: fbguo@uestc.edu.cn; Fax: +86-02883202351; Tel: +86-02883202351

Running title: Minimal bacterial gene set and metabolic network

Supplementary File

File S1| Data describing the 611 genes in the PEG obtained in this work

(Text format, 808KB) http://cefg.uestc.edu.cn/MGS_and_MMN/FileS1.txt

File S2| The SBML (XML) format file for the approximate minimal metabolic network obtained in this work

(SBML format, 523KB) http://cefg.uestc.edu.cn/MGS_and_MMN/FileS2.xml

Supplementary Table

Table S1| The distribution of the 594 re-annotated genes among 22 subsystems

Subsystem	Gene No.
Amino Acids Metabolism and Derivatives	54
Carbohydrates	22
Cell Division and Cell Cycle	8
Cell Wall and Capsule	55
Cofactors, Vitamins, Prosthetic Groups, Pigments	73
DNA Metabolism	29
Dormancy and Sporulation	1
Fatty Acids, Lipids, and Isoprenoids	48
Iron acquisition and metabolism	1
Membrane Transport	10
Metabolism of Aromatic Compounds	1
Miscellaneous	2
Nucleosides and Nucleotides	30
Phosphorus Metabolism	2
Potassium metabolism	7
Protein Metabolism	137
Regulation and Cell signaling	6
Respiration	16
RNA Metabolism	42
Stress Response	9
Sulfur Metabolism	4
Virulence, Disease and Defense	17

Table S2| The minimal gene set obtained in this project [a]

Gene	R[b]	CS[c]	KO	COG
pgsA	13	15	K00995	COG0558I
lig	1	15	K01972	COG0272L
rplE	0	15	K02931	COG0094J
pheT	0	14	K01890	COG0072J
metK	1	14	K00789	COG0192H
thrS	0	14	K01868	COG0441J
serS	0	14	K01875	COG0172J
rpsK	0	14	K02948	COG0100J
rpsG	0	14	K02992	COG0049J
rpsE	0	14	K02988	COG0098J
rpsB	0	14	K02967	COG0052J
rpoD	0	14	K03086	COG0568K

rpmC	0	14	K02904	COG0255J
rplX	0	14	K02895	COG0198J
rplV	0	14	K02890	COG0091J
rplP	0	14	K02878	COG0197J
rplO	0	14	K02876	COG0200J
rplN	0	14	K02874	COG0093J
rplF	0	14	K02933	COG0097J
proS	0	14	K01881	COG0442J
prfA	0	14	K02835	COG0216J
pheS	0	14	K01889	COG0016J
nusA	0	14	K02600	COG0195K
infC	0	14	K02520	COG0290J
infA	0	14	K02518	COG0361J
gyrB	0	14	K02470	COG0187L
ftsZ	0	14	K03531	COG0206D
ftsY	0	14	K03110	COG0552U
dnaG	0	14	K02316	COG0358L
dnaE	0	14	K02316	COG0358L
fabG	38	13	K00059	COG1028IQR
cmk	3	13	K00945	COG0283F
rpoA	0	13	K03040	COG0202K
fabB	19	13	K00647	COG0304IQ
nrdA	4	13	K00525	COG0209F
pyrH	2	13	K09903	COG0528F
ysxC	0	13	K03978	COG0218R
yphC	0	13	K03977	COG1160R
yihA	0	13	K03978	COG0218R
valS	0	13	K01873	COG0525J
tyrS	0	13	K01866	COG0162J
trmD	0	13	K00554	COG0336J
serA	0	13	K03977	COG1160R
secY	0	13	K03076	COG0201U
rpsS	0	13	K02965	COG0185J
rpsQ	0	13	K02961	COG0186J
rpsN	0	13	K02954	COG0199J
rpsC	0	13	K02982	COG0092J
rpoB	0	13	K03043	COG0085K
rplT	0	13	K02887	COG0292J
rplQ	0	13	K02879	COG0203J
rplM	0	13	K02871	COG0102J
rplL	0	13	K02935	COG0222J
rplD	0	13	K02926	COG0088J
pth	0	13	K01056	COG0193J
prlA	0	13	K03076	COG0201U

parE	0	13	K02622	COG0187L
lysU	0	13	K04567	COG1190J
lysS	0	13	K04567	COG1190J
infB	0	13	K02519	COG0532J
frr	0	13	K02838	COG0233J
engB	0	13	K03978	COG0218R
engA	0	13	K03977	COG1160R
alaS	0	13	K01872	COG0013J
gcp	1	12	K01409	COG0533O
ssb	0	12	K03111	COG0629L
secA	0	12	K03070	COG0653U
cysS	0	12	K01883	COG0215J
map	19	12	K01265	COG0024J
cdsA	13	12	K00981	COG0575I
acpP	7	12	K02078	COG0236IQ
adk	6	12	K00939	COG0563F
murG	2	12	K02563	COG0707M
folA	2	12	K00287	COG0262H
prsA	1	12	K00948	COG0462FE
pgk	1	12	K00927	COG0126G
murF	1	12	K01929	COG0770M
murC	1	12	K01924	COG0773M
eno	1	12	K01689	COG0148G
ddlB	1	12	K01921	COG1181M
accA	1	12	K01962	COG0825I
yidC	0	12	K03217	COG0706U
tsf	0	12	K02357	COG0264J
trpS	0	12	K01867	COG0180J
tilS	0	12	K04075	COG0037D
rpsP	0	12	K02959	COG0228J
rpsO	0	12	K02956	COG0184J
rpsM	0	12	K02952	COG0099J
rpsL	0	12	K02950	COG0048J
rpsJ	0	12	K02946	COG0051J
rpsD	0	12	K02986	COG0522J
rpoC	0	12	K03046	COG0086K
rpmI	0	12	K02916	COG0291J
rpmD	0	12	K02907	COG1841J
rpmA	0	12	K02899	COG0211J
rplR	0	12	K02881	COG0256J
rplK	0	12	K02867	COG0080J
rplC	0	12	K02906	COG0087J
rplB	0	12	K02886	COG0090J
rnpA	0	12	K03536	COG0594J

metS	0	12	K01874	COG0143J
metG	0	12	K01874	COG0143J
mesJ	0	12	K04075	COG0037D
ileS	0	12	K01870	COG0060J
holB	0	12	K02341	COG0470L
holA	0	12	K02340	COG1466L
hisS	0	12	K01892	COG0124J
fus	0	12	K02355	COG0480J
fmt	0	12	K00604	COG0223J
era	0	12	K03595	COG1159R
dnaH	0	12	K02341	COG0470L
dnaC	0	12	K02314	COG0305L
dnaB	0	12	K02314	COG0305L
dnaA	0	12	K02313	COG0593L
fabA	19	11	K01716	COG0764I
plsC	16	11	K00655	COG0204I
gmk	3	11	K00942	COG0194F
fbaA	3	11	K01624	COG0191G
tdk	2	11	K00943	COG0125F
pyrG	2	11	K01937	COG0504F
ppa	2	11	K01507	COG1227C,COG0221C
folD	2	11	K01491	COG0190H
tmk	1	11	K00943	COG0125F
murD	1	11	K01925	COG0771M
murA	1	11	K00790	COG0766M
gatB	1	11	K02434	COG0064J
yjeE	0	11	K06925	COG0802R
ydiB	0	11	K06925	COG0802R
rpsR	0	11	K02963	COG0238J
rpsI	0	11	K02996	COG0103J
rpsH	0	11	K02994	COG0096J
rpsF	0	11	K02990	COG0360J
rpmJ	0	11	K02919	COG0257J
rpmH	0	11	K02914	COG0230J
rpmB	0	11	K02902	COG0227J
rplW	0	11	K02892	COG0089J
rplU	0	11	K02888	COG0261J
rplS	0	11	K02884	COG0335J
rimM	0	11	K02860	COG0806J
obg	0	11	K03979	COG0536R
leuS	0	11	K01869	COG0495J
gyrA	0	11	K02469	COG0188L
grpE	0	11	K03687	COG0576O
groS	0	11	K04078	COG0234O

ftsH	0	11	K03798	COG0465O
ftsA	0	11	K03590	COG0849D
ffh	0	11	K03106	COG0541U
dnaX	0	11	K02343	COG2812L
def	0	11	K01462	COG0242J
aspS	0	11	K01876	COG0173J
gapA	2	10	K00134	COG0057G
atpF	1	10	K02109	COG0711C
trmU	0	10	K00566	COG0482J
groL	0	10	K04077	COG0459O
fabD	13	10	K00645	COG0331I
nrdB	4	10	K00526	COG0208F
glmU	3	10	K04042	COG1207M
dfp	3	10	K13038	COG0452H
ribF	2	10	K11753	COG0196H
ppnK	2	10	K00858	COG0061G
nadK	2	10	K00858	COG0061G
mraY	2	10	K01000	COG0472M
tpiA	1	10	K01803	COG0149G
hemH	1	10	K01772	COG0276H
gltX	1	10	K01885	COG0008J
dxs	1	10	K01662	COG1154HI
coaE	1	10	K00859	COG0237H
argS	1	10	K01887	COG0018J
accD	1	10	K01963	COG0777I
ybeY	0	10	K07042	COG0319R
topA	0	10	K03168	COG0551L,COG0550L
secE	0	10	K03073	COG0690U
rpsT	0	10	K02968	COG0268J
rplJ	0	10	K02864	COG0244J
parC	0	10	K02621	COG0188L
ftsW	0	10	K03588	COG0772D
dnaN	0	10	K02338	COG0592L
lpdA	3	9	K00382	COG1249C
fabZ	38	9	K02372	COG0764I
ubiE	3	9	K03183	COG2226H
glyA	2	9	K00600	COG0112E
thyA	1	9	K00560	COG0207F
murI	1	9	K01776	COG0796M
murB	1	9	K00075	COG0812M
ispD	1	9	K00991	COG1211I
iscS	1	9	K04487	COG1104E
guaA	1	9	K01951	COG0518F,COG0519F
gor	1	9	K00383	COG1249C

glmM	1	9	K03431	COG1109G
dapB	1	9	K00215	COG0289E
coaD	1	9	K00954	COG0669H
accC	1	9	K01961	COG0439I
accB	1	9	K02160	COG0511I
yqgF	0	9	K07447	COG0816L
rpmF	0	9	K02911	COG0333J
rpmE	0	9	K02909	COG0254J
prfB	0	9	K02836	COG1186J
nusG	0	9	K02601	COG0250K
lepB	0	9	K03100	COG0681U
ftsQ	0	9	K03589	COG1589M
efp	0	9	K02356	COG0231J
dnaK	0	9	K04043	COG0443O
divIB	0	9	K03589	COG1589M
asnS	0	9	K01893	COG0017J
asnC	0	9	K01893	COG0017J
atpA	0	8	K02111	COG0056C
tuf	0	8	K02358	COG0050J
atpD	0	8	K02112	COG0055C
fabI	26	8	K00208	COG0623I
yneS	16	8	K08591	COG0344S
plsX	16	8	K03621	COG0416I
pssA	13	8	K00998	COG1502I,COG1183I
birA	3	8	K03524	COG0340H
uppS	2	8	K00806	COG0020I
tktA	2	8	K00615	COG0021G
nadD	2	8	K00969	COG1057H
ispU	2	8	K00806	COG0020I
trxA	1	8	K03671	COG0526OC,COG3118O
nadE	1	8	K01916	COG0171H
murE	1	8	K01928	COG0769M
ispF	1	8	K01770	COG0245I
ispE	1	8	K00919	COG1947I
gpmA	1	8	K01834	COG0696G,COG0588G
glmS	1	8	K00820	COG0449M
gatC	1	8	K02435	COG0721J
gatA	1	8	K02433	COG0154J
folB	1	8	K01633	COG1539H
fep	1	8	K02015	COG0609P
dxr	1	8	K00099	COG0743I
dapA	1	8	K01714	COG0329EM
cca	1	8	K00974	COG0617J
acpS	1	8	K00997	COG0736I

<u>*yurV*</u>	0	8	K04488	COG0822C
<u>*waaA*</u>	0	8	K02527	COG1519M
<u>*rpsU*</u>	0	8	K02970	COG0828J
rplA	0	8	K02863	COG0081J
<u>*rbgA*</u>	0	8	K14540	COG1161R
rbfA	0	8	K02834	COG0858J
<u>*nifU*</u>	0	8	K04488	COG0822C
lspA	0	8	K03101	COG0597MU
<u>*lolA*</u>	0	8	K03634	COG2834M
lgt	0	8	K13292	COG0682M
<u>*kdtA*</u>	0	8	K02527	COG1519M
<u>*iscU*</u>	0	8	K04488	COG0822C
glyS	0	8	K01879	COG0751J
<u>*glyQ*</u>	0	8	K01878	COG0752J
glnS	1	8	K01886	COG0008J
atpG	0	8	K02115	COG0224C
msbA	10	7	K11085	COG1132V
<u>*folE*</u>	5	7	K01495	COG0302H
<u>*ispH*</u>	4	7	K03527	COG0761IM
pgi	3	7	K01810	COG0166G
<u>*lytB*</u>	2	7	K03527	COG0761IM
trxB	1	7	K00384	COG0492O
pykA	1	7	K00873	COG0469G
<u>*kdsB*</u>	1	7	K00979	COG1212M
<u>*guaB*</u>	1	7	K00088	COG0516F
<u>*gcpE*</u>	1	7	K03526	COG0821I
<u>*fabH*</u>	29	6	K00648	COG0332I
<u>*cydD*</u>	2	6	K06148	COG4987CO,COG4988CO,COG1132V,COG2274V
<u>*cydC*</u>	2	6	K06148	COG4987CO,COG4988CO,COG1132V,COG2274V
<u>*lpxB*</u>	1	6	K00748	COG0763M
dppD	1	6	K12371	COG0444EP
<u>*dppC*</u>	1	6	K02034	COG1173EP
ddpD	1	6	K12371	COG0444EP
atpC	1	6	K02114	COG0355C
plsB	20	5	K00631	COG2937I
ptsH	6	5	K11189	COG1925G
<u>*ispA*</u>	3	5	K00795	COG0142H
<u>*sucA*</u>	2	5	K01616	COG0567C
potD	1	5	K11069	COG0687E
csdA	1	5	K01766	COG0520E
atpB	1	5	K02108	COG0356C
spoT	3	4	K01139	COG0317TK

gltK	2	4	K10002	COG0765E
nagE	1	4	K02804	COG1263G
fbp	1	4	K03841	COG0158G
aroE	1	4	K00014	COG0169E
ptsI	6	3	K08483	COG1080G
ton	2	3	K03832	COG0810M
purU	1	3	K01433	COG0788F
potC	1	3	K11070	COG1177E
glnP	1	3	K10040	COG0765E
fecA	1	3	K02014	COG4772P,COG1629P
thiS	1	2	K03154	COG2104H
thiI	1	2	K03151	COG0301H
thiGH	1	2	K03149	COG2022H
dppA	1	2	K02035	COG0747E,COG4166E
gltL	2	1	K10004	COG1126E
ydcU	1	3	K02054	COG1176E
ydcT	1	1	K02052	COG3842E
glnH	1	1	K02030	COG0834ET
gcvT	1	1	K00605	COG0404E
fruB	1	1	K00882	COG1105G
fruA	1	1	K02770	COG1299G
dppF	1	3	K12372	COG4608E
aas	6	0		
manZ	3	0		
manY	3	0		
manX	3	0		
gltJ	2	0		
gltI	2	0		
thiF	1	0		
phnN	1	0		
glnQ	1	0		
gcvP	1	0		
gcvH	1	0		
fhuA	1	0		
dppB	1	0		
ddpF	1	0	K12372	COG4608E
ddpC	1	0	K02034	COG1173EP
ddpB	1	0		
ddpA	1	0	K02035	COG0747E,COG4166E
bcp	1	0		
atpE	0	0		COG0636C
atpH	0	0		COG0712C

deaD	0	0		COG0513LKJ
dnaJ	0	2	K03686	COG0484O
greA	0	2	K03624	COG0782K
hpt	0	2	K00760	COG0634F
ksgA	0	2	K02528	COG0030J
pfkA	0	7	K00850	COG0205G
rnc	0	6	K03685	COG0571K
rplI	0	5	K02939	COG0359J
rpmG	0	4	K02913	COG0267J
smpB	0	5	K03664	COG0691O
ung	0	0		COG0692L

[a] Genes with a CS ≥ 8 were obtained by comparative genomic analysis (the first 248 genes); Genes with CS < 8 and the number of reactions associated with > 0 were obtained by gene inactivation in the MMN (immediately following 66 genes); Genes with CS < 8 and the number of reactions associated with = 0 were supplemented by the genes exist in both Koonin *et al.*'s and Gil *et al.*'s MGS (the last 13 genes) Underlined genes were newly identified in our SBGS (107 genes). The genes in blue is the aminoacyl tRNA synthetases.

[b] Indicates the number of reactions associated with a gene.

[c] Indicates the cluster size of a gene.

Table 3|The overlap between the 15 HPEGS14 and HPEGS groups [a]

Group	1	2	3	4	5	6	7	8	9	10	11	12	13	14	15
Coverage [b](%)	94.66	88.57	96.12	90.84	93.58	90.18	92.88	90.84	90.51	95.38	89.86	96.12	92.19	90.18	94.66
Coverage [c](%)	94.35	87.10	95.97	89.92	93.15	89.11	92.34	89.92	89.52	95.16	88.71	95.97	91.53	89.11	94.35

[a]The overlapping gene content of HPEGS14s and HPEGS ranges from 88.57% to 96.25.9% (mean = 91.7%, and variance = 0.0009%). The overlap in HPEGSs number is closer to unity than the gene content, ranging from 88.5% to 96.2% (mean = 92.4%, and variance = 0.0006%). This indicates that the half-retaining strategy is stable so long as the reference species are randomly selected.

[b] The overlap in number between HPEGS and each $HPEGS_{14}$ group.

[c]The overlap in gene content between HPEGS and each $HPEGS_{14}$ group.

Table S4| Bootstrap results for the half-retaining strategy

(XLS format, large table, 65KB) http://cefg.uestc.edu.cn/MGS_and_MMN/TableS4.xls

Table S5| The reactions of the minimal metabolic network

(XLS format, large table, 229KB) http://cefg.uestc.edu.cn/MGS_and_MMN/TableS5.xls

Table S6| The number of covered reactions in each subsystem

Primary subsystem	Secondary subsystem	No.
**Cell envelope biosynthesis**		129
**Lipid biosynthesis and metabolism**		114
	Glycerophospholipid metabolism	71
	Membrane lipid metabolism	43
**Amino acid biosynthesis and metabolism**		9
	Glycine and serine biosynthesis and metabolism	1
	Cysteine biosynthesis and metabolism	1
	Histidine biosynthesis and metabolism	1
	Methionine biosynthesis and metabolism	1
	Threonine and lysine biosynthesis and metabolism	4
	Tyrosine tryptophan and phenylalanine biosynthesis and metabolism	1
**Vitamin biosynthesis and metabolism**		1
**Recovery system**		23
	Lipopolysaccharide biosynthesis Recycling	4
	Recycling of peptidoglycan amino acids | protein degradation	19
**Saccharide biosynthesis and metabolism**		16
	Glycolysis and gluconeogenesis	10
	Pentose phosphate pathway	6
**TCA cycle**		4
**Transport (exchange)**		29
**Cofactor and prosthetic group Biosynthesis**		46
**Folate biosynthesis and metabolism**		4
**Alternate carbon metabolism**		1
**Nucleotide salvage pathway**		20
**Oxidative phosphorylation**		2
**Purine and pyrimidine biosynthesis**		8
**Terpenoids and polyketides metabolism**		2
**tRNA charging**		1
**Other**		22

Table S7| The 13 genes in both Koonin's and Gil's sets but absent in SBGS

Gene	Cluster size	COG	Function
atpE	0	COG0636C	F0F1 ATP synthase subunit C
atpH	0	COG0712C	F0F1 ATP synthase subunit delta
deaD	0	COG0513LKJ	DEAD-box ATP dependent DNA helicase
dnaJ	2	COG0484O	chaperone protein DnaJ
greA	2	COG0782K	transcription elongation factor GreA
hpt	2	COG0634F	hypoxanthine phosphoribosyltransferase
ksgA	2	COG0030J	dimethyladenosine transferase
pfkA	7	COG0205G	6-phosphofructokinase
rnc	6	COG0571K	ribonuclease III
rplI	5	COG0359J	50S ribosomal protein L9
rpmG	4	COG0267J	50S ribosomal protein L33
smpB	5	COG0691O	SsrA-binding protein
ung	0	COG0692L	uracil-DNA glycosylase

Table S8| A list of SBGS genes in that appear in DrugBank and have been validated as drug targets, as well as the genes considered promising targets.

Table S8-1. SBGS genes listed in DrugBank as targets

Gene	Reaction	CS[b]	KO	COG	E-value (Blastp with human protein)
lgt	0	8	K13292	COG0682M	8.20E+00
ispE	1	8	K00919	COG1947I	7.20E+00
fecA	1	3	K02014	COG4772P,COG1629P	7.10E+00
dapB	1	9	K00215	COG0289E	7.00E+00
coaD	1	9	K00954	COG0669H	7.00E+00
murE	1	8	K01928	COG0769M	6.30E+00
ftsZ	0	14	K03531	COG0206D	5.40E+00
murB	1	9	K00075	COG0812M	5.30E+00
fabZ	38	9	K02372	COG0764I	5.10E+00

rpmF	0	9	K02911	COG0333J	4.50E+00
ptsH	6	5	K11189	COG1925G	4.50E+00
rpoA	0	13	K03040	COG0202K	4.40E+00
murF	1	12	K01929	COG0770M	4.40E+00
acpS	1	8	K00997	COG0736I	4.00E+00
trmD	0	13	K00554	COG0336J	3.80E+00
ispF	1	8	K01770	COG0245I	3.70E+00
aroE	1	4	K00014	COG0169E	3.70E+00
fabA	19	11	K01716	COG0764I	3.60E+00
fbaA	3	11	K01624	COG0191G	3.50E+00
dnaN	0	10	K02338	COG0592L	3.50E+00
murD	1	11	K01925	COG0771M	3.50E+00
hemH	1	10	K01772	COG0276H	3.50E+00
murG	2	12	K02563	COG0707M	3.20E+00
rpsT	0	10	K02968	COG0268J	3.10E+00
murC	1	12	K01924	COG0773M	3.00E+00
murA	1	11	K00790	COG0766M	3.00E+00
rpsU	0	8	K02970	COG0828J	2.90E+00
dxr	1	8	K00099	COG0743I	2.60E+00
potD	1	5	K11069	COG0687E	2.20E+00
rpsF	0	11	K02990	COG0360J	2.10E+00
gpmA	1	8	K01834	COG0696G,COG0588G	1.90E+00
fabH	29	6	K00648	COG0332I	1.80E+00
rplJ	0	10	K02864	COG0244J	1.60E+00
folB	1	8	K01633	COG1539H	1.60E+00
murI	1	9	K01776	COG0796M	1.50E+00
lig	1	15	K01972	COG0272L	1.40E+00
nadD	2	8	K00969	COG1057H	8.90E-01
lepB	0	9	K03100	COG0681U	7.80E-01
rpsQ	0	13	K02961	COG0186J	7.00E-01
fhuA	1	0			6.80E-01
ispH	4	7	K03527	COG0761IM	6.70E-01
rpsD	0	12	K02986	COG0522J	6.10E-01
ppa	2	11	K01507	COG1227C,COG0221C	5.90E-01
tmk	1	11	K00943	COG0125F	4.20E-01
tdk	2	11	K00943	COG0125F	4.20E-01
guaA	1	9	K01951	COG0518F,COG0519F	3.50E-01
ssb	0	12	K03111	COG0629L	3.30E-01
pheT	0	14	K01890	COG0072J	2.80E-01
cmk	3	13	K00945	COG0283F	1.50E-01
rpsH	0	11	K02994	COG0096J	1.10E-01
rpsR	0	11	K02963	COG0238J	6.20E-02
acpP	7	12	K02078	COG0236IQ	4.00E-02

atpF	1	10	K02109	COG0711C	2.20E-02
rplP	0	14	K02878	COG0197J	6.00E-03
rpsN	0	13	K02954	COG0199J	5.00E-03
rpsJ	0	12	K02946	COG0051J	4.00E-03
rpsP	0	12	K02959	COG0228J	3.00E-03
nadE	1	8	K01916	COG0171H	3.00E-03
rplV	0	14	K02890	COG0091J	2.00E-03
def	0	11	K01462	COG0242J	2.00E-03
kdsB	1	7	K00979	COG1212M	2.00E-03
rpsS	0	13	K02965	COG0185J	4.00E-17
aas	6	0			2.00E-22
rpsO	0	12	K02956	COG0184J	6.00E-24
rpmA	0	12	K02899	COG0211J	3.00E-26
rpsM	0	12	K02952	COG0099J	5.00E-30
ton	2	3	K03832	COG0810M	1.00E-31
rpsK	0	14	K02948	COG0100J	1.00E-32
rpsL	0	12	K02950	COG0048J	1.00E-33
birA	3	8	K03524	COG0340H	5.00E-38
rpsI	0	11	K02996	COG0103J	1.00E-40
rpsG	0	14	K02992	COG0049J	3.00E-44
rplK	0	12	K02867	COG0080J	3.00E-44
serA	0	13	K03977	COG1160R	4.00E-46
rplD	0	13	K02926	COG0088J	4.00E-48
rpsC	0	13	K02982	COG0092J	8.00E-51
rpsE	0	14	K02988	COG0098J	3.00E-51
folA	2	12	K00287	COG0262H	2.00E-51
pth	0	13	K01056	COG0193J	4.00E-54
gmk	3	11	K00942	COG0194F	1.00E-57
engB	0	13	K03978	COG0218R	5.00E-58
fbp	1	4	K03841	COG0158G	5.00E-58
frr	0	13	K02838	COG0233J	2.00E-58
coaE	1	10	K00859	COG0237H	3.00E-63
rpsB	0	14	K02967	COG0052J	6.00E-64
fabI	26	8	K00208	COG0623I	2.00E-65
parC	0	10	K02621	COG0188L	5.00E-67
adk	6	12	K00939	COG0563F	6.00E-68
trxB	1	7	K00384	COG0492O	3.00E-69
fabG	38	13	K00059	COG1028IQR	4.00E-70
cca	1	8	K00974	COG0617J	2.00E-71
ispA	3	5	K00795	COG0142H	3.00E-72
folE	5	7	K01495	COG0302H	2.00E-75
dnaX	0	11	K02343	COG2812L	3.00E-78
rplC	0	12	K02906	COG0087J	4.00E-80
tsf	0	12	K02357	COG0264J	2.00E-81

pheS	0	14	K01889	COG0016J	1.00E-82
proS	0	14	K01881	COG0442J	1.00E-83
ispD	1	9	K00991	COG1211I	3.00E-85
era	0	12	K03595	COG1159R	2.00E-86
pgi	3	7	K01810	COG0166G	6.00E-88
uppS	2	8	K00806	COG0020I	3.00E-88
map	19	12	K01265	COG0024J	4.00E-90
rpoB	0	13	K03043	COG0085K	7.00E-92
dapA	1	8	K01714	COG0329EM	4.00E-92
nrdB	4	10	K00526	COG0208F	1.00E-98
obg	0	11	K03979	COG0536R	5.00E-99
gcp	1	12	K01409	COG0533O	3.00E-99
tpiA	1	10	K01803	COG0149G	2.00E-101
fmt	0	12	K00604	COG0223J	2.00E-101
thyA	1	9	K00560	COG0207F	2.00E-104
glmU	3	10	K04042	COG1207M	7.00E-111
trpS	0	12	K01867	COG0180J	5.00E-114
gyrA	0	11	K02469	COG0188L	2.00E-117
hisS	0	12	K01892	COG0124J	2.00E-124
prfA	0	14	K02835	COG0216J	3.00E-128
gcvT	1	1	K00605	COG0404E	1.00E-129
tuf	0	8	K02358	COG0050J	8.00E-130
tyrS	0	13	K01866	COG0162J	7.00E-133
gapA	2	10	K00134	COG0057G	3.00E-139
asnS	0	9	K01893	COG0017J	3.00E-140
lpdA	3	9	K00382	COG1249C	4.00E-143
gor	1	9	K00383	COG1249C	2.00E-149
ffh	0	11	K03106	COG0541U	7.00E-150
glyA	2	9	K00600	COG0112E	9.00E-156
fabB	19	13	K00647	COG0304IQ	7.00E-158
pgk	1	12	K00927	COG0126G	3.00E-164
accC	1	9	K01961	COG0439I	3.00E-171
metG	0	12	K01874	COG0143J	1.00E-172
metK	1	14	K00789	COG0192H	3.00E-177
rpoC	0	12	K03046	COG0086K	7.00E-180
topA	0	10	K03168	COG0551L,COG0550L	0.00E+00
thrS	0	14	K01868	COG0441J	0.00E+00
parE	0	13	K02622	COG0187L	0.00E+00
ileS	0	12	K01870	COG0060J	0.00E+00
gyrB	0	14	K02470	COG0187L	0.00E+00
fus	0	12	K02355	COG0480J	0.00E+00
eno	1	12	K01689	COG0148G	0.00E+00
aspS	0	11	K01876	COG0173J	0.00E+00
alaS	0	13	K01872	COG0013J	0.00E+00

tktA	2	8	K00615	COG0021G	0.00E+00
guaB	1	7	K00088	COG0516F	0.00E+00
glmS	1	8	K00820	COG0449M	0.00E+00

Table S8-2. SBGS genes that are promising targets

**Gene**	**Reaction**	**CS[b]**	**KO**	**COG**	**E-value (Blastp with human protein)**
gltK	2	4	K10002	COG0765E	no hit
gltJ	2	0			no hit
ddpC	1	0	K02034	COG1173EP	no hit
rpmD	0	12	K02907	COG1841J	8.70E+00
rpmI	0	12	K02916	COG0291J	8.60E+00
pyrH	2	13	K09903	COG0528F	8.00E+00
rpmE	0	9	K02909	COG0254J	7.00E+00
dnaE	0	14	K02316	COG0358L	7.00E+00
rpmB	0	11	K02902	COG0227J	6.70E+00
holA	0	12	K02340	COG1466L	6.70E+00
glyQ	0	8	K01878	COG0752J	6.50E+00
efp	0	9	K02356	COG0231J	6.40E+00
secE	0	10	K03073	COG0690U	6.00E+00
rpmC	0	14	K02904	COG0255J	5.80E+00
infA	0	14	K02518	COG0361J	5.80E+00
rimM	0	11	K02860	COG0806J	5.60E+00
fep	1	8	K02015	COG0609P	5.60E+00
ftsW	0	10	K03588	COG0772D	5.30E+00
dnaG	0	14	K02316	COG0358L	5.20E+00
ddpA	1	0	K02035	COG0747E,COG4166E	4.80E+00
manY	3	0			4.40E+00
yneS	16	8	K08591	COG0344S	4.30E+00
rpmG	0	4	K02913	COG0267J	4.10E+00
dppC	1	6	K02034	COG1173EP	3.90E+00
glnP	1	3	K10040	COG0765E	3.90E+00
ftsQ	0	9	K03589	COG1589M	3.90E+00
divIB	0	9	K03589	COG1589M	3.90E+00
rnpA	0	12	K03536	COG0594J	3.70E+00
rplR	0	12	K02881	COG0256J	3.60E+00
lspA	0	8	K03101	COG0597MU	3.50E+00
waaA	0	8	K02527	COG1519M	3.40E+00
kdtA	0	8	K02527	COG1519M	3.40E+00
lpxB	1	6	K00748	COG0763M	3.20E+00
manX	3	0			3.20E+00
ribF	2	10	K11753	COG0196H	3.10E+00

plsX	16	8	K03621	COG0416I	3.10E+00
gcpE	1	7	K03526	COG0821I	3.10E+00
lolA	0	8	K03634	COG2834M	2.80E+00
rbfA	0	8	K02834	COG0858J	2.70E+00
nusG	0	9	K02601	COG0250K	2.60E+00
ftsA	0	11	K03590	COG0849D	2.50E+00
gatC	1	8	K02435	COG0721J	2.40E+00
potC	1	3	K11070	COG1177E	2.10E+00
infC	0	14	K02520	COG0290J	2.10E+00
yqgF	0	9	K07447	COG0816L	2.00E+00
dnaA	0	12	K02313	COG0593L	2.00E+00
nagE	1	4	K02804	COG1263G	1.90E+00
holB	0	12	K02341	COG0470L	1.70E+00
dnaH	0	12	K02341	COG0470L	1.70E+00
ddpB	1	0			1.70E+00
tilS	0	12	K04075	COG0037D	1.40E+00
secY	0	13	K03076	COG0201U	1.40E+00
prlA	0	13	K03076	COG0201U	1.40E+00
mesJ	0	12	K04075	COG0037D	1.40E+00
yjeE	0	11	K06925	COG0802R	1.40E+00
ydiB	0	11	K06925	COG0802R	1.40E+00
manZ	3	0			1.30E+00
rplF	0	14	K02933	COG0097J	1.20E+00
glyS	0	8	K01879	COG0751J	1.10E+00
nusA	0	14	K02600	COG0195K	1.00E+00
dnaC	0	12	K02314	COG0305L	1.00E+00
dnaB	0	12	K02314	COG0305L	1.00E+00
thiS	1	2	K03154	COG2104H	9.70E-01
rplU	0	11	K02888	COG0261J	9.60E-01
trxA	1	8	K03671	COG0526OC,COG3118O	8.20E-01
thiGH	1	2	K03149	COG2022H	7.70E-01
pgsA	13	15	K00995	COG0558I	7.10E-01
lytB	2	7	K03527	COG0761IM	6.70E-01
rplA	0	8	K02863	COG0081J	6.40E-01
yurV	0	8	K04488	COG0822C	5.70E-01
nifU	0	8	K04488	COG0822C	5.70E-01
iscU	0	8	K04488	COG0822C	5.70E-01
fruA	1	1	K02770	COG1299G	5.60E-01
ptsI	6	3	K08483	COG1080G	5.10E-01
rplW	0	11	K02892	COG0089J	4.90E-01
groS	0	11	K04078	COG0234O	4.80E-01
atpB	1	5	K02108	COG0356C	4.60E-01
secA	0	12	K03070	COG0653U	4.10E-01
rpmJ	0	11	K02919	COG0257J	3.80E-01

gltI	2	0			3.70E-01
rpoD	0	14	K03086	COG0568K	3.60E-01
thiI	1	2	K03151	COG0301H	3.50E-01
atpC	1	6	K02114	COG0355C	3.00E-01
dppA	1	2	K02035	COG0747E,COG4166E	2.60E-01
rpmH	0	11	K02914	COG0230J	2.40E-01
yidC	0	12	K03217	COG0706U	2.20E-01
ppnK	2	10	K00858	COG0061G	1.60E-01
nadK	2	10	K00858	COG0061G	1.60E-01
dppB	1	0			1.00E-01
smpB	0	5	K03664	COG0691O	9.40E-02
ddlB	1	12	K01921	COG1181M	8.80E-02
rplX	0	14	K02895	COG0198J	4.80E-02
glnH	1	1	K02030	COG0834ET	4.30E-02
greA	0	2	K03624	COG0782K	3.70E-02
plsC	16	11	K00655	COG0204I	2.00E-02
ydcU	1	1	K02054	COG1176E	1.80E-02
phnN	1	0			1.80E-02
mraY	2	10	K01000	COG0472M	1.10E-02
ybeY	0	10	K07042	COG0319R	4.00E-03
rplO	0	14	K02876	COG0200J	3.00E-03
pssA	13	8	K00998	COG1502I,COG1183I	2.00E-03
accB	1	9	K02160	COG0511I	2.00E-03
bcp	1	0			6.00E-10
glnQ	1	0			2.00E-17
rplL	0	13	K02935	COG0222J	7.00E-20
gcvH	1	0			1.00E-22
fruB	1	1	K00882	COG1105G	2.00E-23
ddpF	1	0	K12372	COG4608E	2.00E-23
rplS	0	11	K02884	COG0335J	8.00E-25
rplN	0	14	K02874	COG0093J	5.00E-28
cdsA	13	12	K00981	COG0575I	4.00E-29
rplT	0	13	K02887	COG0292J	5.00E-31
thiF	1	0			5.00E-35
rplQ	0	13	K02879	COG0203J	2.00E-37
rplE	0	15	K02931	COG0094J	2.00E-37
spoT	3	4	K01139	COG0317TK	4.00E-42
rplM	0	13	K02871	COG0102J	6.00E-43
rplI	0	5	K02939	COG0359J	1.00E-45
yphC	0	13	K03977	COG1160R	8.00E-46
engA	0	13	K03977	COG1160R	8.00E-46
accA	1	12	K01962	COG0825I	5.00E-50
ysxC	0	13	K03978	COG0218R	3.00E-51
yihA	0	13	K03978	COG0218R	3.00E-51

grpE	0	11	K03687	COG0576O	3.00E-51
trmU	0	10	K00566	COG0482J	1.00E-55
atpH	0	0		COG0712C	1.00E-57
dppD	1	6	K12371	COG0444EP	4.00E-60
ddpD	1	6	K12371	COG0444EP	4.00E-60
rplB	0	12	K02886	COG0090J	6.00E-65
gltL	2	1	K10004	COG1126E	7.00E-68
accD	1	10	K01963	COG0777I	1.00E-68
rbgA	0	8	K14540	COG1161R	6.00E-69
dfp	3	10	K13038	COG0452H	5.00E-71
hpt	0	2	K00760	COG0634F	8.00E-73
ftsY	0	14	K03110	COG0552U	5.00E-73
purU	1	3	K01433	COG0788F	3.00E-73
dppF	1	1	K12372	COG4608E	2.00E-76
ubiE	3	9	K03183	COG2226H	1.00E-81
ispU	2	8	K00806	COG0020I	3.00E-88
rnc	0	6	K03685	COG0571K	3.00E-88
ksgA	0	2	K02528	COG0030J	2.00E-88
deaD	0	0		COG0513LKJ	2.00E-88
ydcT	1	3	K02052	COG3842E	2.00E-89
atpG	0	8	K02115	COG0224C	3.00E-93
glmM	1	9	K03431	COG1109G	9.00E-96
fabD	13	10	K00645	COG0331I	3.00E-97
prfB	0	9	K02836	COG1186J	3.00E-100
folD	2	11	K01491	COG0190H	2.00E-101
cysS	0	12	K01883	COG0215J	1.00E-110
csdA	1	5	K01766	COG0520E	4.00E-119
cydD	2	6	K06148	COG4987CO,COG4988CO,COG1132V,COG2274V	1.00E-119
cydC	2	6	K06148	COG4987CO,COG4988CO,COG1132V,COG2274V	1.00E-119
dnaJ	0	2	K03686	COG0484O	1.00E-122
prsA	1	12	K00948	COG0462FE	2.00E-129
iscS	1	9	K04487	COG1104E	1.00E-133
gltX	1	10	K01885	COG0008J	8.00E-134
pfkA	0	7	K00850	COG0205G	1.00E-137
serS	0	14	K01875	COG0172J	2.00E-138
asnC	0	9	K01893	COG0017J	3.00E-140
msbA	10	7	K11085	COG1132V	7.00E-143
groL	0	10	K04077	COG0459O	1.00E-144
argS	1	10	K01887	COG0018J	2.00E-147
gatA	1	8	K02433	COG0154J	5.00E-149
plsB	20	5	K00631	COG2937I	2.00E-149
lysU	0	13	K04567	COG1190J	8.00E-152
lysS	0	13	K04567	COG1190J	8.00E-152
atpA	0	8	K02111	COG0056C	3.00E-153

atpD	0	8	K02112	COG0055C	9.00E-158
infB	0	13	K02519	COG0532J	1.00E-164
dxs	1	10	K01662	COG1154HI	3.00E-169
metS	0	12	K01874	COG0143J	1.00E-172
ung	0	0		COG0692L	1.00E-175
atpE	0	0		COG0636C	2.00E-176
ftsH	0	11	K03798	COG0465O	1.00E-179
valS	0	13	K01873	COG0525J	0.00E+00
pykA	1	7	K00873	COG0469G	0.00E+00
nrdA	4	13	K00525	COG0209F	0.00E+00
leuS	0	11	K01869	COG0495J	0.00E+00
gatB	1	11	K02434	COG0064J	0.00E+00
dnaK	0	9	K04043	COG0443O	0.00E+00
sucA	2	5	K01616	COG0567C	0.00E+00
pyrG	2	11	K01937	COG0504F	0.00E+00
glnS	1	8	K01886	COG0008J	0.00E+00
gcvP	1	0			0.00E+00

Table S9| Genes of the SBGS that are not present in *M. genitalium*

Gene	COG	Gene	COG	Gene	COG
fabG	COG1028IQR	*dapA*	COG0329EM	*ftsA*	COG0849D
fabZ	COG0764I	*dapB*	COG0289E	*ftsQ*	COG1589M
fabH	COG0332I	*ddlB*	COG1181M	*ftsW*	COG0772D
fabI	COG0623I	*dppA*	COG0747E,COG4166E	*glyQ*	COG0752J
plsB	COG2937I	*dxr*	COG0743I	*glyS*	COG0751J
fabA	COG0764I	*dxs*	COG1154HI	*kdtA*	COG1519M
fabB	COG0304IQ	*fbp*	COG0158G	*lepB*	COG0681U
fabD	COG0331I	*fecA*	COG4772P,COG1629P	*lolA*	COG2834M
pssA	COG1502I,COG1183I	*fep*	COG0609P	*prfB*	COG1186J
folE	COG0302H	*folB*	COG1539H	*rimM*	COG0806J
ispH	COG0761IM	*gcpE*	COG0821I	*rpmD*	COG1841J
birA	COG0340H	*gcvT*	COG0404E	*waaA*	COG1519M
dfp	COG0452H	*glmS*	COG0449M	*ydiB*	COG0802R
glmU	COG1207M	*glnH*	COG0834ET	*yjeE*	COG0802R
ispA	COG0142H	*glnP*	COG0765E	*aas*	
ubiE	COG2226H	*guaB*	COG0516F	*manZ*	
gltK	COG0765E	*hemH*	COG0276H	*manY*	
gltL	COG1126E	*iscS*	COG1104E	*manX*	
ispU	COG0020I	*ispE*	COG1947I	*gltJ*	
lytB	COG0761IM	*kdsB*	COG1212M	*gltI*	
mraY	COG0472M	*lpxB*	COG0763M	*thiF*	
murG	COG0707M	*murA*	COG0766M	*phnN*	
pyrG	COG0504F	*murB*	COG0812M	*glnQ*	
sucA	COG0567C	*murC*	COG0773M	*gcvP*	
ton	COG0810M	*murD*	COG0771M	*gcvH*	
uppS	COG0020I	*murE*	COG0769M	*fhuA*	
accA	COG0825I	*murF*	COG0770M	*dppB*	
accB	COG0511I	*murI*	COG0796M	*ddpF*	COG4608E
accC	COG0439I	*nagE*	COG1263G	*ddpC*	COG1173EP
accD	COG0777I	*purU*	COG0788F	*ddpB*	
aroE	COG0169E	*thiGH*	COG2022H	*ddpA*	COG0747E,COG4166E
cca	COG0617J	*thiS*	COG2104H	*bcp*	
coaD	COG0669H	*divIB*	COG1589M		
rplI	COG0359J	*smpB*	COG0691O		

Table S10| *M. genitalium* genes omitted from the SBGS

Gene	COG	Gene	COG	Gene	COG
MG_097	COG0692L	MG_068	-	MG_290	COG1120PH
MG_104	COG0557K	MG_069	COG2190G	MG_291	COG3639P
MG_112	COG0036G	MG_074	-	MG_294	COG2211G
MG_118	COG1087M	MG_075	-	MG_296	-
MG_119	COG3845R	MG_076	-	MG_298	COG1196D
MG_120	COG4603R	MG_095	-	MG_302	COG0619P
MG_125	COG0561R	MG_096	-	MG_303	COG1122P
MG_127	COG1393P	MG_101	COG2188K	MG_304	COG1122P
MG_138	COG0481M	MG_103	COG1481S	MG_306	COG0392S
MG_180	COG1122P	MG_476	COG1314U	MG_307	-
MG_182	COG0101J	MG_105	COG1624S	MG_470	COG1192D
MG_187	COG3839G	MG_108	COG0631T	MG_309	-
MG_206	COG0322L	MG_109	COG0515RTKL	MG_310	COG0596R
MG_209	COG0564J	MG_110	COG1162R	MG_312	-
MG_061	-	MG_115	COG1546R	MG_313	-
MG_221	COG2001S	MG_117	-	MG_314	-
MG_238	COG0544O	MG_121	COG1079R	MG_316	-
MG_239	COG0466O	MG_123	-	MG_317	-
MG_244	COG0210L	MG_129	COG1264G	MG_318	-
MG_245	COG0212H	MG_130	COG1418R	MG_319	-
MG_251	COG0423J	MG_132	COG0537FGR	MG_320	-
MG_252	COG0566J	MG_133	-	MG_321	-
MG_259	COG2890J	MG_134	COG0718S	MG_323	COG0569P
MG_261	COG0587L	MG_135	-	MG_515	-
MG_262	COG0258L	MG_137	COG0562M	MG_324	COG0006E
MG_265	COG0561R	MG_139	COG0595R	MG_326	COG1307S
MG_268	COG1428F	MG_140	COG1112L	MG_327	COG0596R
MG_270	COG0095H	MG_477	COG2740K	MG_328	COG1196D
MG_272	COG0508C	MG_144	-	MG_331	-
MG_273	COG0022C	MG_146	COG1253R	MG_516	COG3763S
MG_274	COG1071C	MG_147	-	MG_517	COG0463M
MG_275	COG0446R	MG_148	COG4487S	MG_338	-
MG_276	COG0503F	MG_149	-	MG_342	COG0431R
MG_064	-	MG_478	-	MG_343	-
MG_293	COG0584C	MG_179	COG1122P	MG_344	COG0596R
MG_299	COG0280C	MG_181	COG0619P	MG_348	-
MG_322	COG0168P	MG_183	COG1164E	MG_349	COG3611L
MG_325	COG0267J	MG_184	-	MG_350	-
MG_332	COG0217S	MG_185	-	MG_521	-
MG_339	COG0468L	MG_186	COG1525L	MG_352	COG3331R
MG_346	COG0219J	MG_188	COG1175G	MG_354	-

MG_347	COG0220R	MG_189	COG0395G	MG_356	COG0510M
MG_353	COG0776L	MG_190	COG0618R	MG_360	COG0389L
MG_355	COG0542O	MG_191	-	MG_364	-
MG_357	COG0282C	MG_192	-	MG_366	-
MG_358	COG0632L	MG_200	COG0484O	MG_369	COG1461R
MG_359	COG2255L	MG_202	-	MG_370	COG0564J
MG_367	COG0571K	MG_205	COG1420K	MG_371	COG0618R
MG_379	COG0445D	MG_207	COG0622R	MG_373	-
MG_380	COG0357M	MG_208	COG1214O	MG_374	-
MG_382	COG0572F	MG_480	-	MG_376	-
MG_391	COG0260E	MG_211	-	MG_377	-
MG_067	-	MG_213	COG1354S	MG_381	-
MG_289	-	MG_214	COG1386K	MG_524	-
MG_406	-	MG_217	-	MG_385	COG0584C
MG_408	COG0225O	MG_218	COG1196D	MG_386	-
MG_410	COG1117P	MG_491	-	MG_389	-
MG_411	COG0573P	MG_219	-	MG_395	-
MG_421	COG0178L	MG_220	-	MG_396	COG0698G
MG_425	COG0513LKJ	MG_223	-	MG_397	-
MG_448	COG0229O	MG_225	COG0531E	MG_409	COG0704P
MG_453	COG1210M	MG_226	COG0531E	MG_412	COG0226P
MG_526	COG1136V	MG_230	COG1780F	MG_414	-
MG_285	-	MG_233	COG2868J	MG_525	-
MG_469	COG0593L	MG_235	COG0648L	MG_422	-
MG_286	-	MG_236	COG0735P	MG_423	COG0595R
MG_011	COG0189HJ	MG_237	-	MG_427	COG1765O
MG_018	COG0553KL	MG_241	-	MG_428	COG2197TK
MG_020	COG0596R	MG_242	-	MG_432	COG1284S
MG_022	COG3343K	MG_246	COG1692S	MG_438	COG0732V
MG_025	COG0463M	MG_248	COG2384R	MG_439	-
MG_027	COG0781K	MG_255	-	MG_440	-
MG_028	-	MG_256	-	MG_441	-
MG_029	COG0693R	MG_260	-	MG_443	COG1284S
MG_031	COG2176L	MG_498	COG0266L	MG_447	COG0534V
MG_032	-	MG_263	COG0561R	MG_450	COG1307S
MG_037	COG1488H	MG_267	-	MG_452	-
MG_039	COG0579R	MG_269	COG1196D	MG_454	COG1764O
MG_040	COG1744R	MG_277	-	MG_456	-
MG_051	COG0213F	MG_279	-	MG_460	COG0039C
MG_473	COG0267J	MG_280	-	MG_461	COG1078R
MG_474	-	MG_281	-	MG_467	COG1136V
MG_057	COG1658L	MG_284	-	MG_468	-

Table S11| Genes of a hypothetical artificial cell generated by semi-*de novo* synthesis based on our SBGS and the genome of *M. genitalium*

MG_001	MG_083	MG_159	MG_224	MG_351	MG_446	MG_419	fabB	dxs	glyS
MG_003	MG_086	MG_160	MG_227	MG_361	MG_451	MG_429	fabD	fbp	kdtA
MG_004	MG_087	MG_161	MG_228	MG_362	MG_455	MG_442	pssA	fecA	lepB
MG_005	MG_088	MG_162	MG_229	MG_363	MG_457	MG_459	folE	fep	lolA
MG_006	MG_089	MG_163	MG_231	MG_365	MG_462	MG_464	ispH	folB	prfB
MG_013	MG_090	MG_164	MG_232	MG_375	MG_465	MG_034	birA	gcpE	rimM
MG_015	MG_091	MG_165	MG_234	MG_378	MG_466	MG_085	dfp	gcvT	rpmD
MG_021	MG_092	MG_166	MG_247	MG_383	MG_007	MG_481	glmU	glmS	waaA
MG_023	MG_094	MG_167	MG_249	MG_384	MG_010	MG_240	ispA	glnH	ydiB
MG_026	MG_102	MG_168	MG_250	MG_387	MG_014	MG_008	ubiE	glnP	yjeE
MG_030	MG_106	MG_169	MG_253	MG_392	MG_041	MG_009	gltK	guaB	aas
MG_035	MG_107	MG_170	MG_254	MG_393	MG_046	MG_012	gltL	hemH	manZ
MG_036	MG_111	MG_171	MG_257	MG_394	MG_055	MG_019	ispU	iscS	manY
MG_042	MG_113	MG_172	MG_258	MG_398	MG_062	MG_024	lytB	ispE	manX
MG_043	MG_114	MG_173	MG_266	MG_399	MG_084	MG_033	mraY	kdsB	gltJ
MG_044	MG_122	MG_174	MG_271	MG_400	MG_098	MG_038	murG	lpxB	gltI
MG_045	MG_124	MG_175	MG_278	MG_401	MG_099	MG_049	pyrG	murA	thiF
MG_047	MG_126	MG_176	MG_283	MG_403	MG_100	MG_050	sucA	murB	phnN
MG_048	MG_136	MG_177	MG_287	MG_405	MG_116	MG_052	ton	murC	glnQ
MG_053	MG_141	MG_178	MG_292	MG_407	MG_128	MG_056	uppS	murD	gcvP
MG_054	MG_142	MG_194	MG_295	MG_417	MG_482	MG_059	accA	murE	gcvH
MG_058	MG_143	MG_195	MG_297	MG_418	MG_264	MG_060	accB	murF	fhuA
MG_063	MG_145	MG_196	MG_300	MG_424	MG_505	MG_065	accC	murI	dppB
MG_066	MG_150	MG_197	MG_301	MG_426	MG_311	MG_071	accD	nagE	ddpF
MG_070	MG_151	MG_198	MG_305	MG_430	MG_315	MG_390	aroE	purU	ddpC
MG_072	MG_152	MG_201	MG_330	MG_431	MG_329	MG_445	cca	thiGH	ddpB
MG_077	MG_153	MG_203	MG_334	MG_433	MG_337	fabG	coaD	thiS	ddpA
MG_078	MG_154	MG_204	MG_335	MG_434	MG_522	fabZ	dapA	divIB	bcp
MG_079	MG_155	MG_210	MG_336	MG_435	MG_368	fabH	dapB	ftsA	glyQ
MG_080	MG_156	MG_212	MG_340	MG_437	MG_372	fabI	ddlB	ftsQ	dxr
MG_081	MG_157	MG_216	MG_341	MG_444	MG_388	plsB	dppA	ftsW	fabA
MG_082	MG_158	MG_222	MG_345	rplI	smpB				

Table 12|. The source of essential gene data for the 15 species in the CEG database

**Organisms**	**Experimental method**	**Essential genes** [N]	**Reference**
Acinetobacter baylyi	Single-gene deletion mutants	499	[1]
Bacillus subtilis 168	Systematic gene inactivation	271	[2]
Escherichia coli	Genetic footprinting.Systematic gene inactivation	712	[3, 4]
Francisella novicida U112	Transposon mutant library	392	[5]
Haemophilus influenzae	High-density transposon mutagenesis	642	[6]
Helicobacter pylori	Microarray tracking of transposon mutants	323	[7]
Mycobacterium tuberculosis	High-density transposon mutagenesis	614	[8]
Mycoplasma genitalium	Global transposon mutagenesis	381	[9]
Mycoplasma pulmonis UAB CTIP	Transposon mutagenesis	310	[10]
Pseudomonas aeruginosa	Transposon insertion mutants	335	[11]
Salmonella enterica serovar Typhi	Transposon mutagenesis	353	[12]
Salmonella typhimurium	Insertion-duplication mutagenesis	230	[13]
Staphylococcus aureus NCTC 8325	Transposon-mediated differential hybridisation	351	[14]
Streptococcus pneumoniae	Targeted disruption.Allelic replacement mutagenesis	244	[15, 16]
Vibrio cholera N16961	Near-saturating transposon insertion library.	779	[17]

[N] : means the number of essential genes.

Reference

1. de Berardinis V, Vallenet D, Castelli V, Besnard M, Pinet A, Cruaud C, Samair S, Lechaplais C, Gyapay G, Richez C, et al: **A complete collection of single-gene deletion mutants of Acinetobacter baylyi ADP1.** *Mol Syst Biol* 2008, **4:**174.
2. Kobayashi K, Ehrlich SD, Albertini A, Amati G, Andersen KK, Arnaud M, Asai K, Ashikaga S, Aymerich S, Bessieres P, et al: **Essential Bacillus subtilis genes.** *Proc Natl Acad Sci U S A* 2003, **100:**4678-4683.
3. Gerdes SY, Scholle MD, Campbell JW, Balazsi G, Ravasz E, Daugherty MD, Somera AL, Kyrpides NC, Anderson I, Gelfand MS, et al: **Experimental determination and system level analysis of essential genes in Escherichia coli MG1655.** *J Bacteriol* 2003, **185:**5673-5684.
4. Baba T, Ara T, Hasegawa M, Takai Y, Okumura Y, Baba M, Datsenko KA, Tomita M, Wanner BL, Mori H: **Construction of Escherichia coli K-12 in-frame, single-gene knockout mutants: the Keio collection.** *Mol Syst Biol* 2006, **2:**2006 0008.
5. Gallagher LA, Ramage E, Jacobs MA, Kaul R, Brittnacher M, Manoil C: **A comprehensive transposon mutant library of Francisella novicida, a bioweapon surrogate.** *Proc Natl*

Acad Sci U S A 2007, **104:**1009-1014.
6. Akerley BJ, Rubin EJ, Novick VL, Amaya K, Judson N, Mekalanos JJ: **A genome-scale analysis for identification of genes required for growth or survival of Haemophilus influenzae.** *Proc Natl Acad Sci U S A* 2002, **99:**966-971.
7. Salama NR, Shepherd B, Falkow S: **Global transposon mutagenesis and essential gene analysis of Helicobacter pylori.** *Journal of bacteriology* 2004, **186:**7926-7935.
8. Sassetti CM, Boyd DH, Rubin EJ: **Genes required for mycobacterial growth defined by high density mutagenesis.** *Mol Microbiol* 2003, **48:**77-84.
9. Glass JI: **Essential genes of a minimal bacterium.** *Proceedings of the National Academy of Sciences* 2006, **103:**425-430.
10. French CT, Lao P, Loraine AE, Matthews BT, Yu H, Dybvig K: **Large-scale transposon mutagenesis of Mycoplasma pulmonis.** *Mol Microbiol* 2008, **69:**67-76.
11. Liberati NT, Urbach JM, Miyata S, Lee DG, Drenkard E, Wu G, Villanueva J, Wei T, Ausubel FM: **An ordered, nonredundant library of Pseudomonas aeruginosa strain PA14 transposon insertion mutants.** *Proc Natl Acad Sci U S A* 2006, **103:**2833-2838.
12. Langridge GC, Phan M-D, Turner DJ, Perkins TT, Parts L, Haase J, Charles I, Maskell DJ, Peters SE, Dougan G: **Simultaneous assay of every Salmonella Typhi gene using one million transposon mutants.** *Genome research* 2009, **19:**2308-2316.
13. Knuth K, Niesalla H, Hueck CJ, Fuchs TM: **Large-scale identification of essential Salmonella genes by trapping lethal insertions.** *Mol Microbiol* 2004, **51:**1729-1744.
14. Chaudhuri RR, Allen AG, Owen PJ, Shalom G, Stone K, Harrison M, Burgis TA, Lockyer M, Garcia-Lara J, Foster SJ: **Comprehensive identification of essential Staphylococcus aureus genes using Transposon-Mediated Differential Hybridisation (TMDH).** *BMC Genomics* 2009, **10:**291.
15. Thanassi JA, Hartman-Neumann SL, Dougherty TJ, Dougherty BA, Pucci MJ: **Identification of 113 conserved essential genes using a high-throughput gene disruption system in Streptococcus pneumoniae.** *Nucleic Acids Res* 2002, **30:**3152-3162.
16. Song JH, Ko KS, Lee JY, Baek JY, Oh WS, Yoon HS, Jeong JY, Chun J: **Identification of essential genes in Streptococcus pneumoniae by allelic replacement mutagenesis.** *Mol Cells* 2005, **19:**365-374.
17. Cameron DE, Urbach JM, Mekalanos JJ: **A defined transposon mutant library and its use in identifying motility genes in Vibrio cholerae.** *Proc Natl Acad Sci U S A* 2008, **105:**8736-8741.

Supplementary Figure

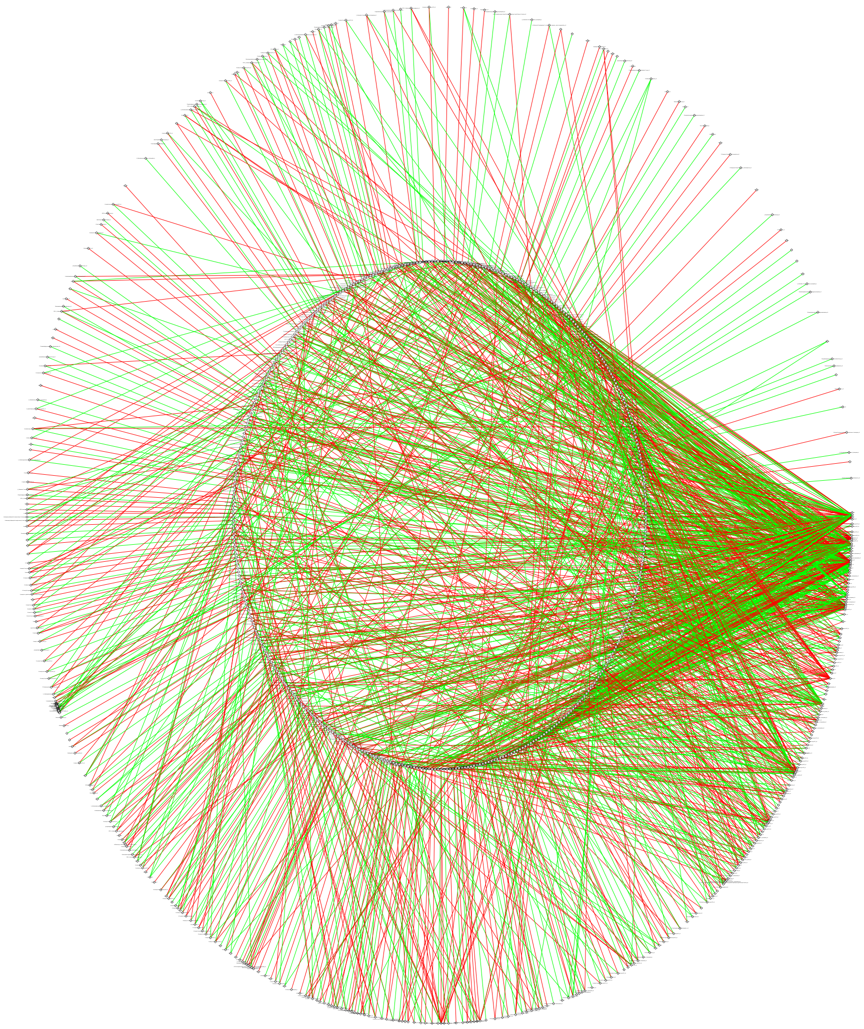

Figure S1. An outline of the approximate minimal metabolic network（vectorgraph）

The detailed metabolic map of each sub-system can be observed in http://cefg.uestc.edu.cn/MGS_and_MMN/FigureSub.pdf

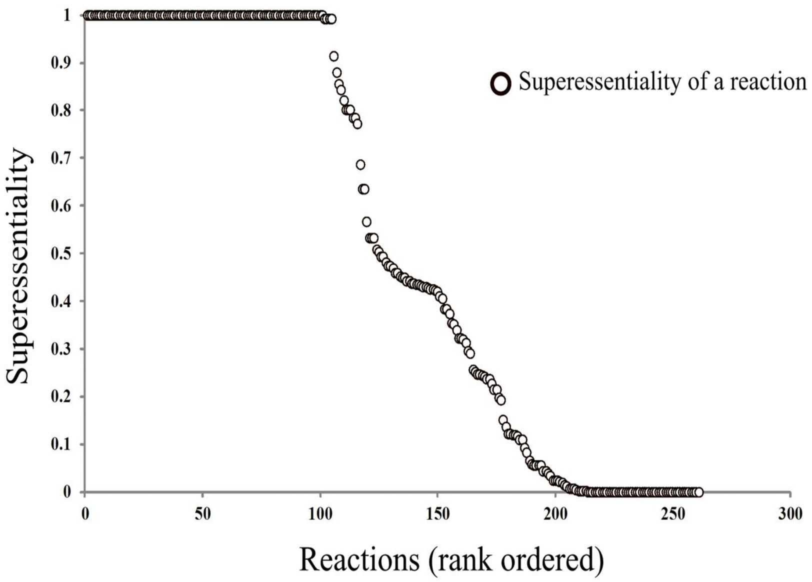

Figure S2. Distribution of the super-essentiality values of reactions in the MMN

The reactions are ranked in descending order according to super-essentiality. The vertical axis represents the super-essentiality of the reactions, while the horizontal axis indicates the number of reactions with the corresponding super-essentiality value.
